# Integrated stress response of *Escherichia coli* to methylglyoxal: transcriptional readthrough from the *nemRA* operon enhances protection through increased expression of glyoxalase I

**DOI:** 10.1111/mmi.12234

**Published:** 2013-05-05

**Authors:** Ertan Ozyamak, Camila Almeida, Alessandro P S de Moura, Samantha Miller, Ian R Booth

**Affiliations:** 1 School of Medical Sciences, Institute of Medical Sciences, University of AberdeenAberdeen, AB25 2ZD, UK; 2 Institute of Complex Systems & Mathematical Biology, School of Natural & Computer Sciences, University of AberdeenAberdeen, AB24 3UE, UK

## Abstract

Methylglyoxal (MG) elicits activation of K^+^ efflux systems to protect cells against the toxicity of the electrophile. ChIP-chip targeting RNA polymerase, supported by a range of other biochemical measurements and mutant creation, was used to identify genes transcribed in response to MG and which complement this rapid response. The SOS DNA repair regulon is induced at cytotoxic levels of MG, even when exposure to MG is transient. Glyoxalase I alone among the core MG protective systems is induced in response to MG exposure. Increased expression is an indirect consequence of induction of the upstream *nemRA* operon, encoding an enzyme system that itself does not contribute to MG detoxification. Moreover, this induction, via *nemRA* only occurs when cells are exposed to growth inhibitory concentrations of MG. We show that the *kdpFABCDE* genes are induced and that this expression occurs as a result of depletion of cytoplasmic K^+^ consequent upon activation of the KefGB K^+^ efflux system. Finally, our analysis suggests that the transcriptional changes in response to MG are a culmination of the damage to DNA and proteins, but that some integrate specific functions, such as DNA repair, to augment the allosteric activation of the main protective system, KefGB.

## Introduction

Bacterial adaptation blends both modulation of cytoplasmic enzymes and changes in gene expression to effect a response that enhances survival of changes in the environment. The bacterial response to electrophiles has been well-characterized at the level of activation of protective K^+^ efflux systems (Ferguson, 1999), but studies of the contribution from specific transcriptional events are more limited. Methylglyoxal (MG) is a toxic electrophile produced during unbalanced sugar metabolism in *Escherichia coli* (*E. coli*) and other bacteria (Freedberg *et al*., 1971; Russell, 1993). Conservation of the glyoxalase system for MG detoxification from bacteria to man suggests that such exposure is common to all lifestyles (Mannervik, 2008; Sukdeo and Honek, 2008; Suttisansanee and Honek, 2011). Several studies hint towards the production of MG in macrophages in response to the entry of pathogenic microorganisms such as *Salmonella* or *Mycobacterium*, as part of the host defence mechanisms (Eskra *et al*., 2001; Eriksson *et al*., 2003; Rachman *et al*., 2006). The occurrence of MG in many food and beverage products has also been reported (Nemet *et al*., 2006; Tan *et al*., 2008) and this may contribute to background levels of DNA damage (Kenyon and Walker, 1980; Sassanfar and Roberts, 1990; Yuan *et al*., 2008) since MG is a known mutagen (Marnett *et al*., 1985; Dorado *et al*., 1992). Recently, exposure to MG has been suggested to underpin the faster rate of development of ‘persister’ cells in *E. coli* populations (Girgis *et al*., 2012), which may reflect the mutagenic potential of this electrophile.

**Fig. 1:**
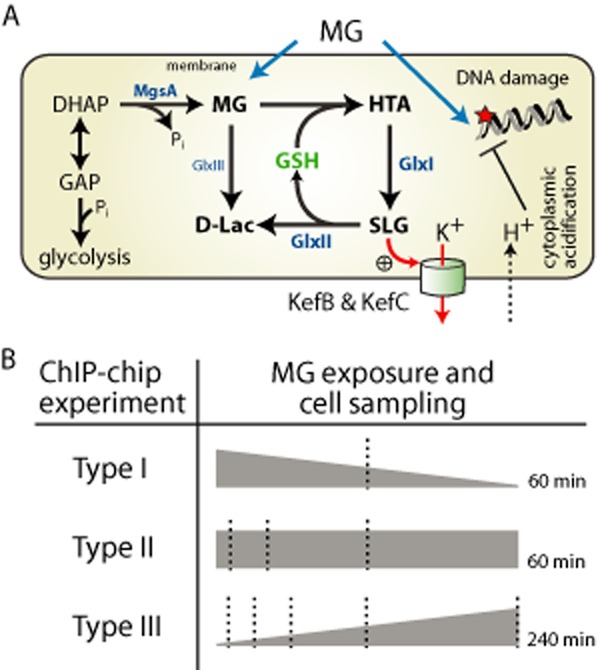
MG stress in *E. coli* and ChIP-chip analysis. A. Routes of MG exposure and protective systems in *E. coli*. B. Experimental design of ChIP-chip analysis upon MG stress. Dotted lines indicate time points of sampling.

In enteric bacteria, the major route for MG production is from dihydroxyacetone phosphate (DHAP), which is converted to MG by the action of MG synthase (*mgsA*; Fig. 1A) (Hopper and Cooper, 1971). During normal growth the production of MG is maintained at a low level by the requirement for homotropic activation of MgsA by DHAP and by the strong inhibition of the enzyme by free phosphate. Consequently, MG production only occurs at a high rate when the cellular pool of phosphate is depleted and DHAP pools are very high – such conditions arise when cells move from famine to feast, a condition that predisposes cells to perform high levels of transport and metabolism of sugars (Totemeyer *et al*., 1998). Low concentrations of MG are bacteriostatic, but at high levels MG kills bacteria via covalent modification of proteins, DNA and lipids (Krymkiewicz, 1973; Colanduoni and Villafranca, 1985). MG modifies bases in DNA (Krymkiewicz, 1973), particularly guanine, and repair can lead to double strand breaks in the DNA (Ferguson *et al*., 2000) and induction of DNA repair enzymes (Kenyon and Walker, 1980; Sassanfar and Roberts, 1990; Yuan *et al*., 2008).

Protection against MG in *E. coli*, and other enteric bacteria, has several components. A central feature is the formation of cysteinyl adducts with glutathione (GSH) and the subsequent metabolism by the GSH-dependent glyoxalase system, encoded by the unlinked *gloA* and *gloB* genes (Ferguson *et al*., 1998; MacLean *et al*., 1998; Kizil *et al*., 2000). This pathway leads to cytoplasmic recycling of GSH during MG breakdown, in contrast to the fate of other electrophile adducts formed with GSH and/or other protective thiols, such as mycothiol (Ferguson *et al*., 1993; 1995; Ferguson and Booth, 1998; Eskra *et al*., 2001; Fahey, 2001; Newton *et al*., 2009; 2012). In addition, *E. coli* has evolved a more sophisticated protective mechanism that involves both GSH-dependent and GSH-independent enzyme systems and K^+^ efflux (KefGB and KefFC) systems that respond directly to GSH and GSH adducts (GSX) (Elmore *et al*., 1990; Ferguson *et al*., 1995; MacLean *et al*., 1998; Ozyamak *et al*., 2010). The GSH-dependent glyoxalase system, consisting of glyoxalase I and II (GlxI & GlxII), provides the main route for MG detoxification resulting in the production of d-lactate (Fig. 1A) (MacLean *et al*., 1998; Mannervik, 2008). Survival is highly dependent on the activity of these enzyme systems via their impact on *S*-lactoylglutathione (SLG) pools (Ozyamak *et al*., 2010). The balance of the activities of GlxI & GlxII determines the cytoplasmic pool of SLG, which is the activator of ligand-gated K^+^ efflux systems KefGB and KefFC (Fig. 1A). Activation of KefGB and KefFC causes cytoplasmic acidification, the degree of which is directly correlated with survival (Ozyamak *et al*., 2010). Although *E. coli* has two systems, KefGB and KefFC, of which the former is dominant in the response to MG, many Gram-negative bacteria have a single Kef system. A third, GSH-independent, enzyme (glyoxalase III, GlxIII) with the ability to convert MG directly to d-lactate has been identified as Hsp31 (encoded by *hchA*) (Misra *et al*., 1995; Subedi *et al*., 2011). In addition, a number of Aldo-keto reductases may play ancillary roles in metabolizing MG, via their activity as low specificity, aldehyde reductase (Ko *et al*., 2005; Lee *et al*., 2010).

In this study we applied ChIP-chip technology (Grainger *et al*., 2005; 2009) to measure changes in the genome-wide redistribution of RNA polymerase (RNAP) during MG stress. ChIP-chip directly measures the occupation of DNA by specific binding proteins (Herring *et al*., 2005). When RNAP is targeted, as here, one may infer changes in transcriptional patterns analogous to classical transcriptomics studies (Grainger *et al*., 2005). In addition to avoiding problems with mRNA stability, additional information is gained from the RNAP distribution across the transcribed regions, such as in the case of stalled RNAP molecules (Wade *et al*., 2007; Grainger and Busby, 2008). In this study these analyses of transcription were complemented by biochemical analyses and mutant creation to test specific hypotheses arising from the observed patterns of RNAP distribution. Our study provides the first insight into the transcriptional response of *E. coli* to sudden exposure to either sublethal or lethal concentrations of MG and also describes the temporal response as the MG concentration increases progressively during unbalanced metabolism. A large number of transcriptional changes were observed in response to MG exposure, but of these only the enhanced expression of the *gloA* gene, encoding GlxI, and the SOS response are directly beneficial. Other changes are either neutral or counter-protective. The expression data are consistent with transcriptional responses responding primarily to cell damage rather than activation of a regulon of protective systems.

## Results

### Experimental design

The response of cell populations to MG depends both on the MG concentration and on the cell density (Fraval and McBrien, 1980). We have performed ChIP-chip with DNA-RNAP complexes isolated from *E. coli* MG1655 cells incubated under three different growth regimes (Fig. 1B): (I) sublethal concentration of MG (0.8 mM MG at cell density OD_650_ ∼ 0.4). In these experiments the MG concentration falls progressively throughout the experiment due to detoxification by the cells; (II) lethal dose of MG (0.8 mM at OD_650_ ∼ 0.04). Here the MG concentration falls very slowly throughout the experiment, but remains at a lethal concentration throughout the sampling period; and (III) progressive intoxication (cells synthesize MG throughout the experiment, such that the concentration rises from zero to > 0.7 mM over a 4 h time period) (Totemeyer *et al*., 1998) (for more details see *Experimental procedures* and *Supporting information*). In addition, strain MG1655 was compared with derivative MJF632 lacking KefGB and KefFC, the electrophile-activated K^+^ efflux systems that confer protection (Ferguson, 1999).

### Exposure to sublethal concentrations of MG (Type I)

**Fig. 2:**
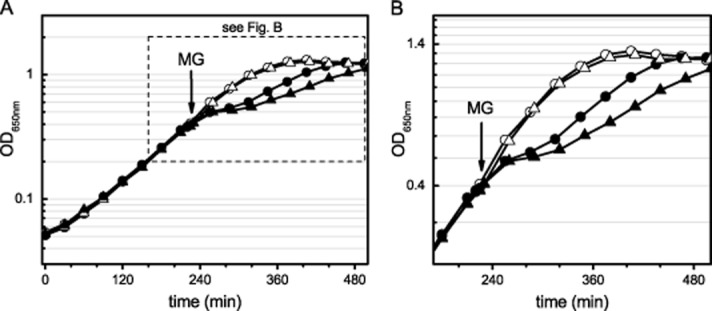
Growth of *E. coli* strains MG1655 and MJF632 (Δ*kefGB*, Δ*kefFC*) before and after exposure to a sublethal MG concentration during mid-exponential phase. (A) Strain MG1655 (circles) and MJF632 (triangles) were grown in K_0.2_ minimal medium overnight and diluted into fresh medium to an OD_650_ ∼ 0.05. Two parallel cultures were inoculated for each strain: a control culture (open circles or triangles) and a test culture (filled circles or triangles). Cells were grown to OD_650_ ∼ 0.4 and the test culture was treated with 0.8 mM MG (indicated by arrow) and the growth was assessed further. Three independent experiments were performed and representative data are shown. A section of the data in (A) is presented enlarged in (B).

**Fig. 3:**
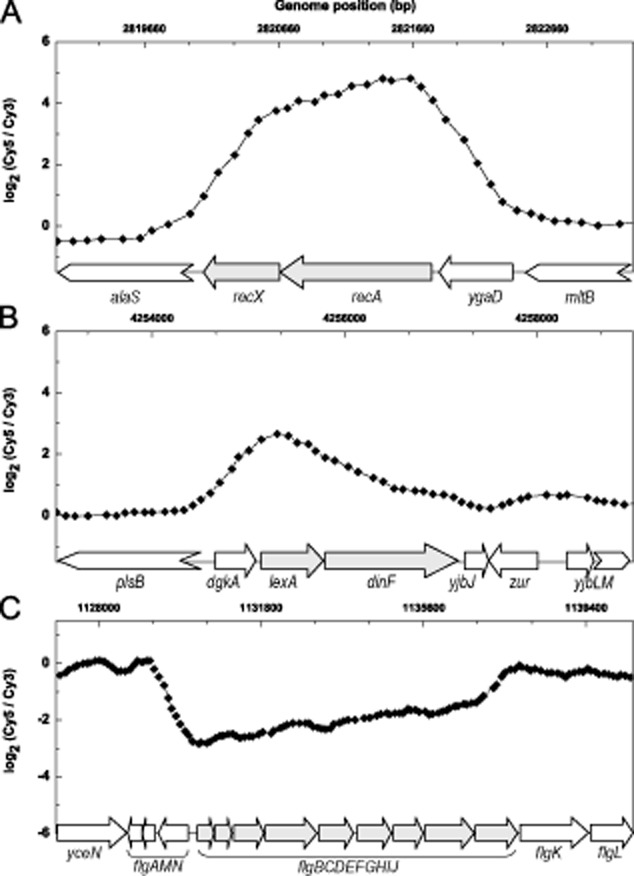
RNAP occupancy in *E. coli* MG1655 is increased across LexA-regulated genes *recAX* (A) and *lexA-dinF* (B) and decreased across *flg* genes (C) upon sublethal MG challenge (Type I experiment). Immunoprecipitated DNA from MG-treated and untreated cells was labelled with Cy5 and Cy3 respectively. Four independent experiments were performed. Data were smoothed using adjacent averaging over 5 data points. Genes and their transcriptional orientation are indicated as arrows. Chevron arrows indicate genes with genomic boundaries beyond the illustration here.

Treatment of mid-exponential phase cultures (initial OD_650_ = 0.4) with 0.8 mM MG led to only approximately 50% growth inhibition, with the implication that transcription should remain active throughout the experimental period (Fig. 2). Treated cells recovered the maximum growth rate 60 min after addition of MG, which corresponds to the time taken to reduce the external concentration of MG to a non-inhibitory concentration (∼ 0.1 mM) (MacLean *et al*., 1998). The culture subsequently reached the same final cell density as non-treated cells (Fig. 2), indicating that no irreversible damage had occurred from this experimental regime. A strain lacking both the KefGB and KefFC K^+^ efflux systems, MJF632, exhibited a delayed recovery from exposure to MG (Fig. 2B); we have previously shown that lack of the efflux systems does not modify the detoxification rate (Ferguson *et al*., 1995; Almeida, 2009). ChIP-chip analysis was performed after 30 min, midway through the period of reduced growth rate. A number of genes were induced and others repressed (Table S2, Dataset S1), as represented by peaks and troughs, respectively, in the data. RNAP peaks across genome areas were in good agreement with the boundaries of known transcription units (TUs) (Fig. 3)*.*

### Induction of the LexA-regulated SOS regulon

**Fig. 4:**
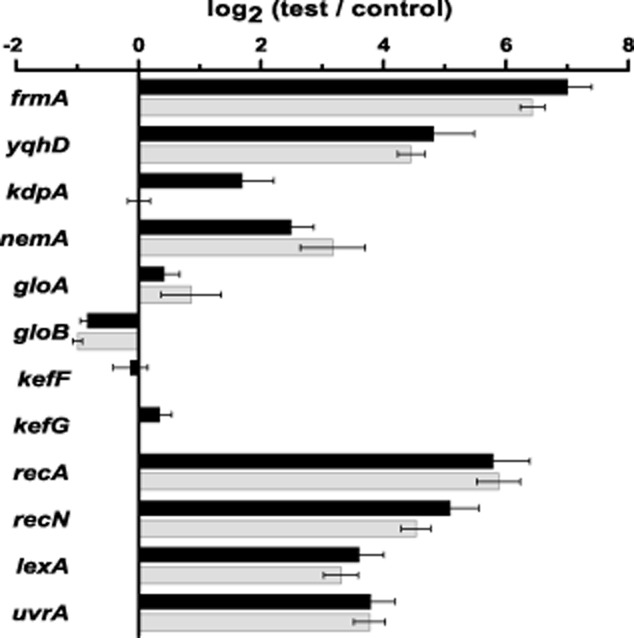
Transcript level changes upon MG stress are similar between strains MG1655 and MJF632, and correlate with changes in RNAP occupancy. Transcript levels for a number of genes in strain MG1655 (black bars) and MJF632 (Δ*kefGB*, Δ*kefFC*; gray bars) were determined by qRT-PCR. Cells were grown and treated exactly as in Type I ChIP-chip experiments. Transcript levels were normalized against the internal control genes *topB*, *trkA* and *polA*. Changes in transcript levels are expressed as fold-changes relative to untreated control samples. Error bars indicate the standard deviation of three independent experiments.

MG is known to cause DNA modification, principally the formation of adducts with deoxyadenosine and deoxyguanine (Papoulis *et al*., 1995; Frischmann *et al*., 2005). It was not surprising, therefore, that the SOS system was induced after MG exposure. LexA-regulated genes (e.g. *recAX*, *lexA*-*dinF*, *dinB*) were among the genes with increased RNAP occupancy (Fig. 3A and B, Table S2), indicating a high transcriptional activity for DNA repair, consistent with the DNA damage expected during MG treatment reported by others (Kenyon and Walker, 1980; Sassanfar and Roberts, 1990; Yuan *et al*., 2008). This transcriptional pattern was confirmed by qRT-PCR, which demonstrated very significant increases in mRNA for SOS genes (Fig. 4). The increased expression of the SOS regulon was in line with expectations and thus provided a good baseline for the other changes in gene expression discussed below. Increased RNAP occupancy at SOS response genes was accompanied by decreased occupancy at genes associated with fast growth, such as those for motility and amino acid biosynthetic pathways (e.g. *flgBCDEFGHIJ* and *gltBD*; Fig. 3C, Dataset S1).

### Induction of the *nemRA* operon is beneficial for MG tolerance through an indirect mechanism

**Fig. 5:**
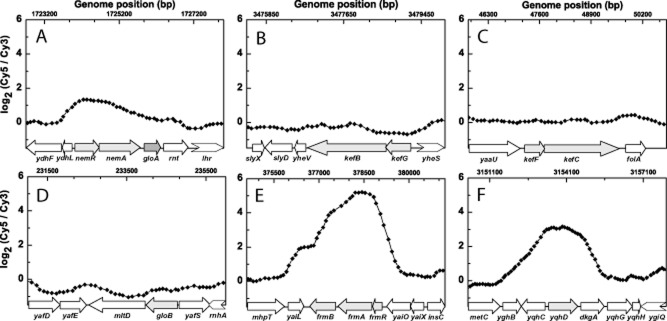
*E. coli* MG1655 induces several detoxification systems upon MG stress, but not core protective systems. RNAP occupancy across genes and operons in cells exposed to a sublethal MG concentration (Type I experiment) is shown. Same data set as in Fig. 3 used for illustration. *nemRA* and *gloA* (A), *kefGB* (B), *kefFC* (C), *gloB* (D), *frmRAB* (E), *yqhD* (F).

We have previously demonstrated the critical role of GlxI in generating the regulator of K^+^ efflux systems KefGB and KefFC (Fig. 1A) and thus mediating protection against MG (MacLean *et al*., 1998; Ozyamak *et al*., 2010). We thus specifically sought to analyse the GlxI-encoding gene *gloA*. Bioinformatic analysis, as provided on the RegulonDB database (Salgado *et al*., 2013), suggested the presence of a potential promoter in the intergenic region between *nemA* and *gloA* indicating that *nemRA* and *gloA* can form two independent TUs (Fig. S1). However, there is no marked transcriptional terminator between *nemA* and *gloA*, suggesting the possibility for transcriptional readthrough. Increased RNAP binding was observed along the length of the *gloA* gene. However, this was continuous with the binding to the upstream *nemRA* operon (Fig. 5A). The *nemRA* operon encodes the *N*-ethylmaleimide reductase, NemA, and NemR, the repressor protein of the *nemRA* operon. It was shown previously that NemR can be inactivated by alkylating reagents such as *N*-ethylmaleimide, but also by MG (Umezawa *et al*., 2008). Thus, we sought to determine whether there was a real linkage between the *nemRA* and *gloA* genes.

**Fig. 6:**
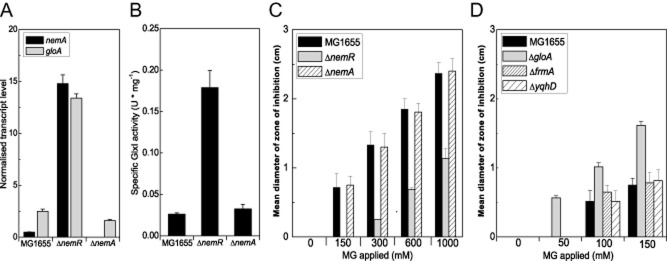
Induction of *nemRA* is beneficial for *gloA* expression. A. Transcript levels for *nemA* (black bars) and *gloA* (light gray bars) in strains MG1655, MJF643 (Δ*nemR*) and MJF644 (Δ*nemA*) as quantified by qRT-PCR. Cells were grown to mid-exponential phase in K_0.2_ medium and total RNA was isolated. B. Specific GlxI activities in cytoplasmic cell extracts of the strains in (A). Cells were grown under conditions matching those to isolate total RNA. Assays were performed using two protein concentrations from each extract to ensure that the enzyme was rate limiting. C. A Δ*nemR* mutant is more resistant to MG than wild type strain MG1655. D. The MG sensitivity of strains MG1655 and MJF637 (Δ*gloA*; positive control) was compared with those of strains MJF635 (Δ*frmA*) and MJF636 (Δ*yqhD*). C and D. MG disc assays were conducted as detailed in *Supporting information*. Each strain was tested using differently concentrated MG solutions on a single K_115_ plate. Error bars indicate the standard deviation of three independent experiments.

A mutant strain deleted for *nemR* was created (see *Supporting information*) and transcription of the *gloA* gene assessed by qRT-PCR of the mRNA pool from cells extracted after exposure to sublethal MG concentrations*.* In wild type cells transcripts for both *nemA* and *gloA* were detected with the latter being more abundant than the former, consistent with independent promoters. Deletion of *nemR* led to 15 and 5-fold higher levels of transcript for *nemA* and *gloA* respectively (Fig. 6A). In contrast, deletion of *nemA* (in a NemR^+^) background did not modify the level of the *gloA* transcript detected. Consistent with these data, we observed that cell-free extracts contained similar levels of GlxI activity whenever the strain was NemR^+^, but GlxI activity was increased ∼ 5-fold in a *nemR* deletion strain, consistent with translation of the more abundant *gloA* mRNA (Fig. 6B). Finally, this increased expression of *gloA* was manifested in a decreased sensitivity to MG in the Δ*nemR* strain compared with both the wild type and the Δ*nemA* strain (these two strains exhibiting equivalent sensitivity) (Fig. 6C). The data are consistent with readthrough from the *nemA* promoter providing enhancement of expression of *gloA.*

Several systems have been proposed to be components of the defence mechanism against MG in *E. coli* (Misra *et al*., 1995; Ferguson *et al*., 1998; Subedi *et al*., 2011). We assessed whether genes for the core protective systems also showed increased transcription as result of sublethal MG exposure. RNAP occupancy across the genes for GlxII (*gloB*) and the K^+^ efflux systems (*kefGB* and *kefFC*) were not significantly changed whether assessed by ChIP-chip (Fig. 5B–D) or by qRT-PCR analysis of mRNA pools (Fig. 4). Moreover, no increased RNAP occupancy was detected for genes of GSH biosynthesis enzymes (*gshA*, *gshB*, *ybdK*) or for GSH-independent GlxIII (*hchA*) (Dataset S1). In contrast, strong induction of the *frmAB* and *yqhD* genes, involved in aldehyde detoxification was observed (Fig. 4, Fig. 5E and F). The GSH-dependent FrmAB enzyme system is involved is the detoxification of formaldehyde (Herring and Blattner, 2004; Gonzalez *et al*., 2006). The YqhD system has been shown to have aldo-keto reductase activity against a wide range of aldehydes, including MG (Lee *et al*., 2010). Despite the increased transcription of genes for these systems, single deletion mutants lacking *frmA* or *yqhD* did not exhibit increased sensitivity to MG, whereas a *gloA* mutant, lacking GlxI, showed the expected sensitivity (Fig. 6D). Recent work has identified that a double mutant lacking both *gloA* and *yqhD* acquired increased sensitivity to glyoxal, but not to MG, when compared with the single *gloA* mutant (Lee *et al*., 2010), thus confirming that increased expression of YqhD is unlikely to be a major factor in MG tolerance. The data show that *E. coli* cells do not induce key protective systems as an adaptation strategy to sublethal MG exposure, but do induce systems that appear not to have a major physiological role for MG tolerance.

### Transcriptional response is rapid in cells exposed to lethal concentrations of MG (Type II)

Exposure of cultures at low cell density (OD_650_ ∼ 0.04) to MG causes rapid cell death (∼ 0.2% cells are viable after 30 min in the presence of 0.8 mM MG) (MacLean *et al*., 1998; Ozyamak *et al*., 2010). We investigated the transcriptional response at intervals (2.5, 10 & 30 min) after MG challenge; (Type II experiment; Fig. 1B) (see *Experimental procedures*, Dataset S1). We observed a very similar RNAP distribution pattern and enrichment ratios to the Type I experiments above (Table S2). Time-dependent changes in the ChIP-chip signals for members of the SOS response genes were observed. In the 2.5 min sample increases in expression of the SOS genes were very small or not reproducible. Stronger signals for the SOS genes were observed in both the 10 min and 30 min samples (Fig. S2A and B, Table S2). A significant difference from cells treated with sublethal MG was the increased RNAP occupancy of genes associated with oxidative stress. The OxyR-regulated *ahpCF* operon was clearly upregulated, with the highest enrichment at 2.5 min and decreased signals thereafter (Fig. S2C). Other OxyR-regulated genes (Storz *et al*., 1990a,b,c) such as *trxC*, *grxA* or *dps* also exhibited this pattern (Fig. S2D–F). No consistent increase in RNAP occupancy across the *ahpCF* operon was observed in Type I experiments (sublethal MG). This difference may reflect transient induction of OxyR-regulated genes upon lethal MG challenge, possibly due to transient GSH depletion, that is missed in Type I experiments due to the very significantly lower MG concentration at the sampling time due to rapid detoxification of MG at the higher cell density (the external concentration would fall to ∼ 0.4 mM) (Almeida, 2009).

### Transcriptional response during progressive MG intoxication (Type III)

**Fig. 7:**
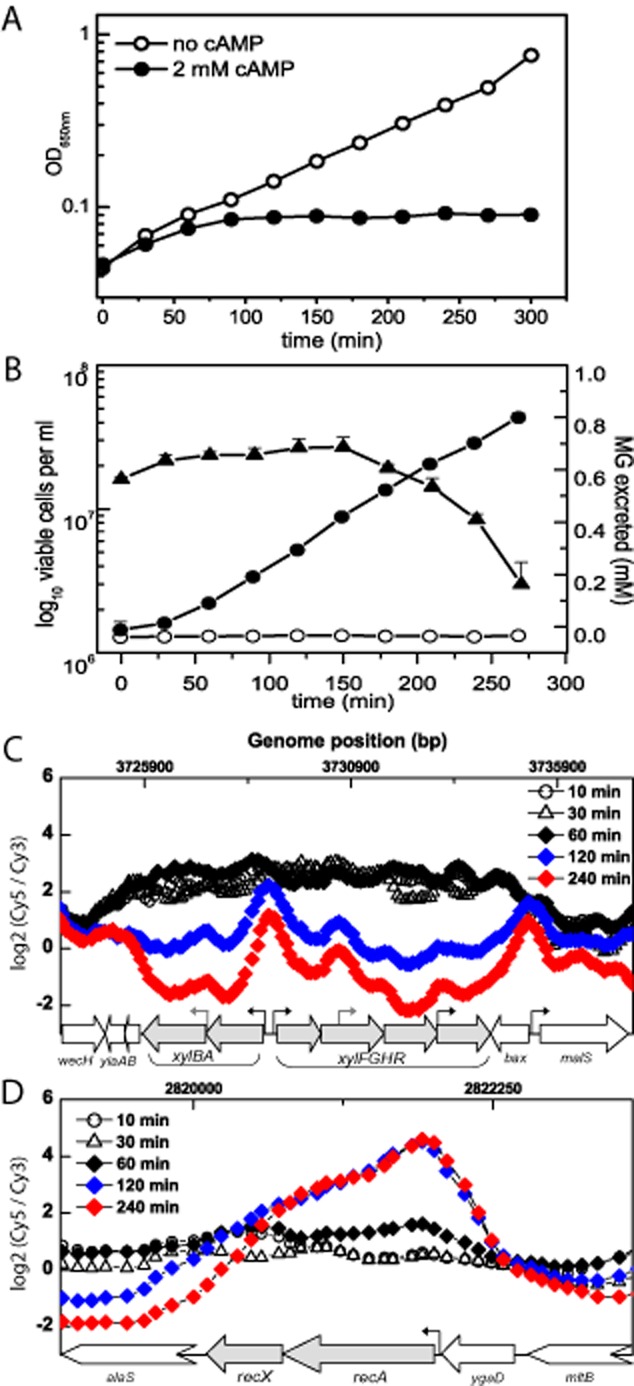
Progressive MG accumulation results in cell death and shift in RNAP occupancy. A. Growth of *E. coli* MG1655 in the absence (open circles) and presence of 2 mM cAMP (filled circles) as grown for Type III experiments. Three independent growth experiments were performed (representative data are shown). B. Cell viability of strain MG1655 (triangles) and MG production in the presence (filled circles) and absence of cAMP (open circles) over the course of the growth experiment shown in Fig. 7. Error bars indicate the standard deviation of three independent experiments. C and D. ChIP-chip signals for the *xyl* genes (C) and *recAX* (D) at different time points after diluting cells into K_0.2_ medium containing cAMP, relative to reference cells (Type III experiments). DNA from cAMP-treated and reference cells were labelled with the Cy5 and Cy3 respectively. Averages from two independent experiments are shown. Black arrows indicate locations of known promoters. Gray arrows indicate promoters predicted by BPROM (http://linux1.softberry.com). Data smoothing and labels are as described in Fig. 3.

**Fig. 8:**
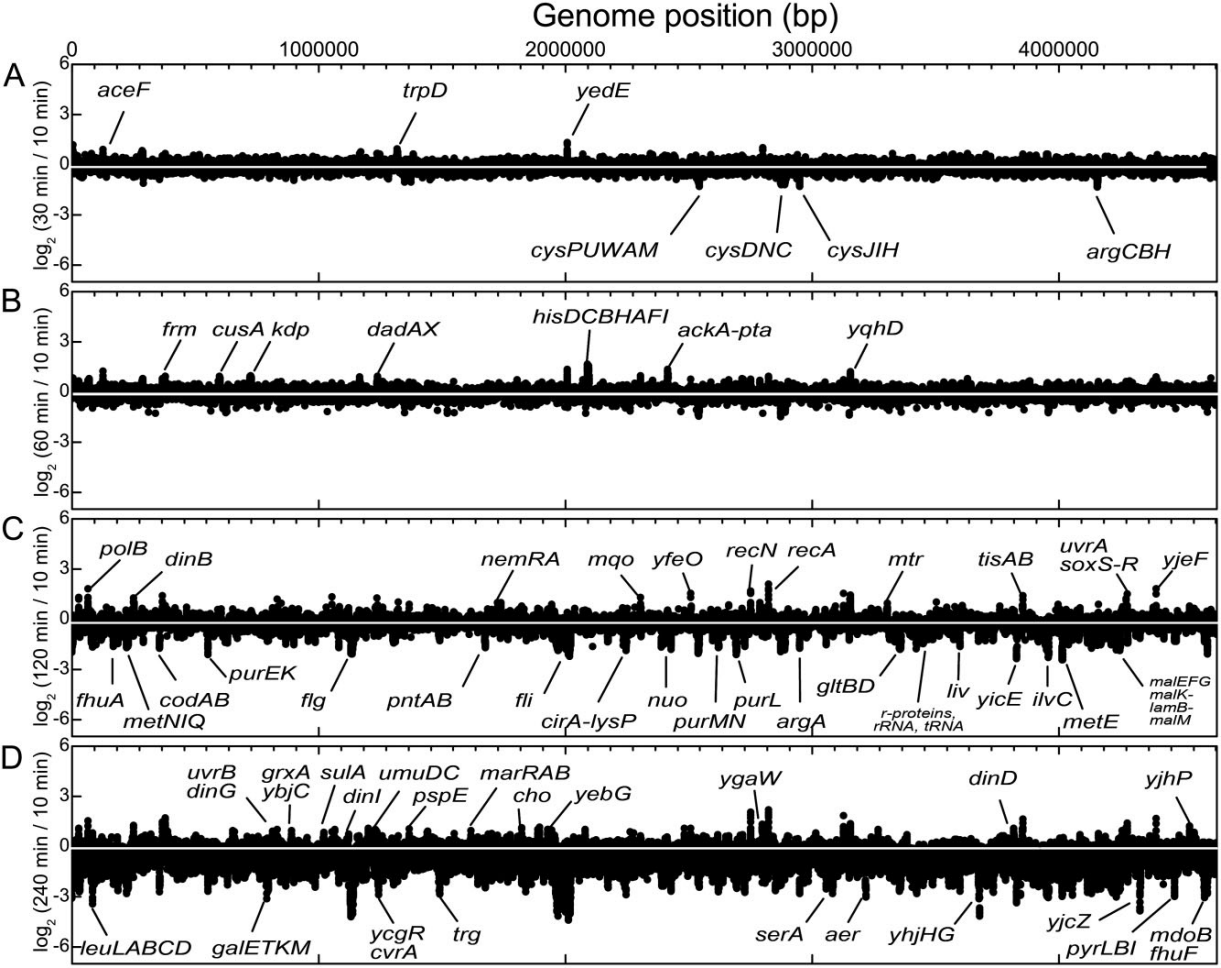
Genome-wide RNAP occupancy changes in MG1655 upon progressive MG accumulation in Type III experiments. ChIP-chip signals for test DNA (cAMP-treated) at *t*_30min_ (A), *t*_60min_ (B), *t*_120min_ (C) and *t*_240min_ (D) was compared *in silico* to test signals at *t*_10min_, thus visually eliminating cAMP-induced changes and highlighting MG induced changes in RNAP occupancy. For each time point Cy5 signal intensities were averaged from two independent replicates and log_2_ ratios were calculated. Log_2_ ratios were then normalized with respect to eight reference regions across the genome that exhibited low signal intensities in both cAMP-treated and untreated cells and that did not change over time (see *Supporting information*). For a complete list of significant changes see Table S3.

Bacteria frequently produce MG as a metabolic by-product during adaptation from famine to feast (Freedberg *et al*., 1971; Totemeyer *et al*., 1998) and consequently sudden exposure of cells to a high concentration of MG may not be physiological. We therefore sought to compare the transcriptional response during the production and accumulation of MG in the medium with the responses described above. In addition, such a regime would provide an indication of the concentration dependence of the response to MG. Previously, we have described the production of MG by *E. coli* cells growing on xylose when stimulated to increased transcription of the xylose regulon by cAMP addition, which mimics the famine to feast scenario (Totemeyer *et al*., 1998). Over a 5 h time-course the growth rate slowed to zero (at ∼ 0.4 mM MG), followed by death as the MG concentration rises to 0.8 mM (Fig. 7A and B). RNAP distribution profiles at each time point were compared either with the control, with no added cAMP (Fig. 7C and D, Dataset S1), or to the initial sample at 10 min after cAMP addition (Fig. 8A–D, Table S3), at which time the level of MG was undetectable. After 30 min incubation with cAMP the MG pool had risen to ∼ 50 μM and only limited changes in gene expression were observed (both induction and repression). Genes for cysteine biosynthesis, which is required for GSH biosynthesis, appeared to be repressed by MG (i.e. RNAP exhibited reduced occupancy at this operon; Fig. 8A). However, since growth continues for at least one further generation, the existing enzymes must remain active at this MG concentration and a potential reduction in transcription may not be significant for cysteine production.

After 60 min (MG concentration 100–150 μM; Fig. 8B, Table S3) the transcription pattern was clearly perturbed with specific enzyme systems being induced, including the *frmAB* and *yqhD* genes (Fig. 8B, Fig. S3C and D, Table S3). However, as mentioned above, these enzymes do not appear to play a major role in protection against MG (Fig. 6D). Induction of the *his* regulon (Fig. 8B) may be explained by the reaction of MG with this amino acid causing partial starvation (Aldini *et al*., 2005) and release from attenuation (Yanofsky, 1981; Barnes and Tuley, 1983). Large-scale changes in the transcriptome were evident at both 120 min and 240 min at which time the MG concentration has reached growth inhibitory (∼ 0.4 mM) and lethal levels (∼ 0.7 mM) respectively. In both cases strong induction of the SOS regulon and *soxSR* and *marRAB* was evident (Fig. 8C and D, Table S3). It was also at this time point that *nemA* (and *gloA*) transcription was also increased (Fig. 8C, Figs S4 and S5B). During the final stages of MG intoxication the overall balance of RNAP binding favoured a few specialist DNA repair functions, while the majority of genes involved in housekeeping metabolism were repressed (Fig. 8C and D). These observations were confirmed by qRT-PCR for selected genes (Fig. S4).

Throughout the time series, genes that are regulated by cAMP exhibited high RNAP occupancy, which is indicative that cAMP remains abundant. At the outset the TUs were uniformly occupied by RNAP (Fig. 7C), but as the MG concentration rose further (Fig. 7B), RNAP was progressively located at the promoter regions and at intergenic regions, producing pronounced peaks and valleys from the previous uniform distribution. For example, the *xylFGHR* (Fig. 7C) and *manXYZ* operons, and *malT* (Fig. S3A and B) were upregulated after 10 min and remained high throughout the experiment despite the modified distribution of RNAP. Thus, there was no general shutdown of the cAMP-CRP regulatory system even at lethal concentrations of MG. Transcription also continued unabated (e.g. *frmA*, *nemA* and *recA*) as revealed by qRT-PCR analysis (Fig. S4), despite the greater polarity in the distribution of RNA polymerase in later time samples.

### The counter-protective Kdp system is induced by MG

**Fig. 9:**
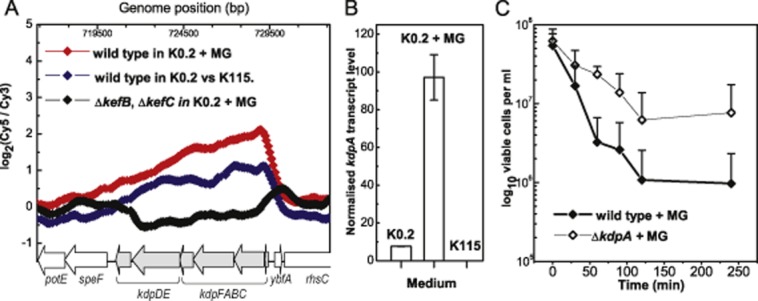
RNAP occupancy across *kdp* operons is affected under different conditions, and *kdp* expression sensitizes cells to MG stress. A. Shown in blue are ChIP-chip data from MG1655 cells, grown to mid-exponential phase, in K_0.2_ medium relative to cells grown in K_115_ medium. Three independent experiments were performed and shown are representative data. Shown in red are ChIP-chip data from MG1655 cells grown in K_0.2_ medium and exposed to a sublethal MG concentration relative to untreated cells during mid-exponential growth (Type I experiment). Shown in black are equivalent data to the ones shown in red except that experiments were performed with strain MJF632 (Δ*kefGB*, Δ*kefFC*). Four independent experiments were performed and shown are representative data. Data smoothing and labels for all ChIP-chip data as in Fig. 3. B. *kdpA* transcript levels in strain MG1655 grown in different minimal media as determined by qRT-PCR. Transcript levels were normalized against the internal control genes *topB*, *trkA* and *polA*. Shown are the averages and standard deviations of three independent experiments. C. Cell viability of strains MG1655 (filled diamonds) and MG1655Δ*kdpA* (open diamonds) in K_0.2_ medium upon MG challenge. Error bars indicate the standard deviation of three independent experiments.

Induction of the *kdpFABC* and *kdpDE* operons was observed in all three sets of ChIP-chip data (i.e. Type I, II & III; Figs 4 and 8B). The *kdpFABC* genes encode a high affinity scavenging P-type K^+^-ATPase (Laimins *et al*., 1978; Rhoads *et al*., 1978). Transcription of the structural genes is under control of KdpDE and this two-component regulatory system responds to insufficiency of the Trk and Kup, constitutive K^+^ transporters, to maintain the K^+^ pool (Laimins *et al*., 1981). During MG stress, the expression of the Kdp system is consistent with the expected enhanced K^+^ loss consequent upon activation of KefGB and KefFC systems. However, it is also counterintuitive since K^+^ loss and consequent cytoplasmic acidification is intrinsic to the mechanisms protecting cells against MG (Ferguson, 1999). Thus, we sought to verify the original ChIP-chip data. Firstly, we established that the signals for the *kdpFABCDE* region responded simply to K^+^ sufficiency by simply exchanging the low K^+^ growth medium (K_0.2_) for high K^+^ (K_115_). When expressed as a ratio (K_0.2_/K_115_) the *kdpFABCDE* genes exhibited increased RNAP occupation relative to the flanking genomic regions (Fig. 9A), consistent with their transcription during steady state growth in low K^+^ medium. The observed changes in occupancy of the *kdpFABCDE* operon by RNAP in low and high K^+^ media were confirmed by qRT-PCR (Fig. 9B). Growth in the presence of MG at low K^+^ resulted in further enhancement of the *kdpFABCDE* ChIP-chip signal, suggesting that K^+^ loss associated with activation of KefGB and KefFC during MG detoxification generated an enhanced signal for transcription (Fig. 9A). To test this prediction we generated ChIP-chip data for strain MJF632 (Δ*kefGB*, Δ*kefFC*), which lacks both K^+^ efflux systems. Consistent with the model there was no increase in ChIP signal for the *kdpFABCDE* operon in this strain (Fig. 9A) and qRT-PCR analysis of mRNA pools confirmed this observation (Fig. 4). Other transcriptional responses to MG were similar to the wild type strain (Fig. 4, Table S2) and ChIP-chip signal patterns and mRNA stability of highly expressed genes were similar (*Supporting information*, Fig. S6). Previously we have established that expression of the *kdpFABCDE* operon sensitizes *E. coli* to MG (Ferguson *et al*., 1996). We sought to verify that the strain used here, MG1655, also dies more rapidly if exposed to MG when the *kdpFABC* operon is active. An isogenic mutant lacking *kdpA*, the K^+^ channel forming subunit, was found to survive exposure to MG ∼ 10-fold better than the wild type (Fig. 9C). Thus, the cells express a system that counters their own survival.

## Discussion

MG toxicity is encountered in all forms of life and the response most frequently utilizes GSH-dependent detoxification of the electrophile and repair of damage by specialist inducible enzyme systems (Ferguson, 1999). *E. coli* offers a paradigm for the bacterial response to MG. Glyoxalase-type enzymes are ubiquitous in bacteria despite the rather more limited distribution of GSH (Suttisansanee and Honek, 2011). This disparity has partially been resolved by the discovery of sugar-based thiol compounds that are intrinsic components of the detoxification system in some Gram-positive bacteria and by the recent elucidation of novel biosynthetic pathways to γ-glutamylcysteine peptides (Newton *et al*., 2009; 2012; Gaballa *et al*., 2010; Suttisansanee and Honek, 2011; Veeravalli *et al*., 2011) in a wide range of organisms. *E. coli* augments the detoxification by a novel acidification mechanism by which cytoplasmic K^+^ is exchanged for external H^+^ via the KefGB and KefFC systems (Ferguson, 1999). The activity of these systems is controlled by the balance between reduced GSH and GSH adducts formed during detoxification. Similar systems have been identified in *Bacillus* (YhaTU; (Booth *et al*., 2003; Fujisawa *et al*., 2004; 2007) and the discovery of bacillithiol, mycothiol and glyoxalases specific for these thiols leaves open the possibility of equivalent regulation of K^+^ efflux (J. Helmann, pers. comm.). The *E. coli* system is so effective that it offers protection even when detoxification is essentially blocked by mutations affecting the second enzyme in the glyoxalase pathway, GlxII (*gloB*) (Ozyamak *et al*., 2010). GlxI, the first enzyme in the pathway is essential for protection against MG because of its central role in generating the activator of KefGB. Thus, simply removal of GSH through formation of the hemithioacetal, the spontaneous reaction product formed by reaction of MG with GSH, is not enough to activate KefGB – the system requires the GlxI-catalysed formation of SLG (MacLean *et al*., 1998; Ozyamak *et al*., 2010). Given that this essentially constitutive, allosterically modulated system is so effective we sought to determine the transcriptional response to MG using ChIP-chip analysis to follow the positioning of RNAP on the genome. The data present a comprehensive picture of the transcriptional response of *E. coli* to MG and reveals intriguing changes in gene expression some of which are counterintuitive.

Even when exposed to lethal concentrations of MG that kill > 99.9% of cells, the bacteria remain transcriptionally active throughout the treatment. Moreover, previous studies reported that even when MG-mediated growth inhibition was maximal, incorporation of external label into RNA and protein continued, albeit at a lower rate (Fraval and McBrien, 1980). No analysis of the balance between rRNA/tRNA and mRNA was performed in that early study. In our study similar RNAP distributions, and inferred transcription patterns, were observed under the three different experimental regimes tested. Genes that are transcribed in response to MG can be ascribed to three broad classes – (i) those required for DNA repair, (ii) enzyme systems that are known to be regulated by proteins that are modulated by the modification of critical cysteine residues, and (iii) systems that appear to be adventitiously expressed as a consequence of the changed physiology of the cells as they detoxify MG. Among the latter is the transient response of the OxyR regulon during sudden exposure to lethal concentrations of MG (Type II experiments, Fig. S2) and *soxRS* genes during Type III progressive intoxication (Fig. 8C). The OxyR response to hydrogen peroxide (H_2_O_2_) is known to be transient (Zheng *et al*., 1998; Aslund *et al*., 1999; Carmel-Harel and Storz, 2000) and would fit the kinetics observed here. Depletion of GSH pools by MG, leading to a transient change in cytoplasmic redox potential, may be sufficient to explain the increased transcription of some of the genes under OxyR control, whereas direct covalent modification of OxyR by MG seems less likely to be the mechanism (Zheng *et al*., 1998). No increased RNAP binding was observed at the genes for GSH biosynthesis that might be expected under conditions of oxidative stress, but this may simply reflect the hierarchy of gene expression with the OxyR regulon (Carmel-Harel and Storz, 2000). In contrast, the increased expression of the SOS regulon is as predicted from the known reaction of MG with DNA causing base modification (Krymkiewicz, 1973; Kenyon and Walker, 1980; Sedgwick and Vaughan, 1991; Ferguson *et al*., 2000; Moolenaar *et al*., 2000; Karschau *et al*., 2011). Type II experiments reveal that this response is moderately slow – increases in *recAX* expression are not seen in the 2.5 min time point after increased exposure (Fig. S2), presumably reflecting the rate at which the balance between excision repair creation of single strand gaps exceeds the rate of re-synthesis of the DNA and ligation (Karschau *et al*., 2011). In Type III experiments it is clear that severe growth inhibition precedes the major induction of the SOS regulon (Fig. 7A and D).

One of the most striking transcriptional responses that *E. coli* cells elicited to MG challenge was the induction of several potential detoxification systems (*nemA*, *frmAB*, *yqhD*) (Fig. 5A, E and F). However, these systems do not appear to have a physiological protective role against MG toxicity since deletion mutants exhibited the same levels of MG tolerance as the wild type strain (Fig. 6C and D). The molecular basis for these transcriptional responses is most probably covalent modification of regulatory proteins by MG. Thus, NemR, the repressor protein of the *nemRA* operon, is rendered inactive by electrophiles (e.g. N-ethylmaleimide and MG) through the modification of at least one specific cysteine residue (Umezawa *et al*., 2008). During the preparation of this manuscript two recent studies have shown that the modification of NemR leads to decreased binding of this protein to the *nemRA* promoter leading to readthrough transcription of *gloA* (Gray *et al*., 2013; Lee *et al*., 2013) in agreement with our independent observations here. Lee *et al*. (2013) report that Cys21 and Cys116 are critical for responding to electrophiles and propose a model in which NemR regulation is mediated by the formation of Cys21–Cys21 and Cys116–Cys116 disulphide bonds on the dimeric protein. Gray *et al*. (2013), who studied the HOCl-responsiveness of NemR conclude that oxidation of Cys106 is sufficient for NemR's ability to respond to bleach (HOCl) and other reactive chlorine species.

Upregulation of the *frmRAB* operon, encoding a formaldehyde detoxification system, under MG stress may also be interpreted in the context of repressor alkylation/modification, since the FrmR protein also contains a conserved cysteine residue. Similarly, transcription of the *yqhD* gene, encoding a non-specific aldo-keto reductase activity, is regulated by YqhC, a cysteine-rich protein encoded upstream of *yqhD* (Fig. 5F) (Lee *et al*., 2010). The *yqhD-dkgA* and *nemRA-gloA* operons can be induced by a diverse range of reactive molecules (Turner *et al*., 2011; Gray *et al*., 2013; Lee *et al*., 2013) supporting the hypothesis that induction of the above mentioned detoxification systems is a general consequence of the electrophilic nature of MG. Studies with *B. subtilis* show that both formaldehyde and MG elicit a stress response characteristic for thiol-reactive, non-aldehyde electrophiles, such as quinones and diamide (Nguyen *et al*., 2009). Moreover, the authors demonstrated an essential role for cysteine modification in the transcriptional regulator, AdhR, in response to formaldehyde and MG. Thus, while some transcriptional responses are undoubtedly protective, others simply reflect the protein damage via cysteine modification.

Our previous work has established that three major variables have the potential to lower the sensitivity to MG: a low activity for the Kdp system, increased expression of both KefGB and GlxI, leading to enhanced potassium efflux and cytoplasmic acidification and ultimately enhanced protection. However, Kdp expression is increased by the presence of MG, which is counter-protective (Ferguson *et al*., 1996) and confirmed here (Fig. 9C). In this study we saw no evidence for increased expression of the KefGB and KefFC systems (Fig. 5B and C) that could have countered the effects of increased Kdp activity. In contrast, increased expression of *gloA* leading to elevated GlxI activity and thus greater activation of KefGB (MacLean *et al*., 1998; Ozyamak *et al*., 2010), can arise by readthrough from the *nemRA* operon as noted above. Although the scale of *gloA* mRNA change and ChIP-chip signals (Figs 4 and 5A respectively) is small, our previous studies have shown that a 30–50% increase in GlxI activity would be sufficient to cause a very large change in survival (MacLean *et al*., 1998).

The gene order *nemRA-gloA* is conserved among the γ-proteobacteria and the ChIP-chip data here suggest that transcriptional readthrough from the *nemRA* operon into *gloA* arises at concentrations of MG that are just sufficient to cause growth inhibition (∼ 0.4 mM) (Fig. 7A, Figs S4 and S5B). The lack of a strong terminator signal between *nemA* and *gloA* provides a mechanism for amplifying the activity of GlxI when cells encounter inhibitory levels of MG. An independent σ^70^ promoter has been predicted to lie 5’ to *gloA* (Fig. S1), which might function to produce the ‘housekeeping’ level of GlxI observed in cells not previously exposed to the electrophile (Fig. 6B). Moreover, the *gloA* gene is expressed from multicopy plasmids lacking the upstream *nemRA* genes, which is consistent with the presence of a functional promoter (MacLean *et al*., 1998). A recent study proposes that the *nemRA-gloA* genes constitute a system for the reduction of quinones and glyoxals, and point towards a similar transcriptional organization in some eukaryotic organism (Lee *et al*., 2013). However, a distinction has to be made between different glyoxals (glyoxal and MG) in terms of cell physiology. In a previous study the authors have shown that YqhD is the major detoxifying enzyme for glyoxal and that the GlxI & II system does not serve as an efficient pathway for its detoxification (Lee *et al*., 2010). Moreover, it is worth noting that it is unknown whether glyoxal elicits the activation the KefGB and KefFC systems as MG does. Interestingly, another study shows that HOCl stress can result in the increased production of MG *E. coli* (Gray *et al*., 2013). The authors suggest the relevance of the *nemRA-gloA* gene organization, regulated by the HOCl-sensitive NemR, to be that cells anticipate the production of MG and induce the protective GlxI enzyme.

Our data highlight the concentration-dependent nature of responses when MG accumulates progressively, and correlate this with the effect on growth and survival. At low MG concentrations (< 0.1 mM, a concentration that only slightly inhibits growth; MacLean *et al*., 1998) a limited number of major changes occurred affecting selected operons (Fig. 8A and B), but wide-ranging changes in gene expression were evident at later time points (MG concentration > 0.4 mM; Fig. 7B), with repression dominating over induction (Fig. 8C and D). The lack of RNAP at these repressed loci cannot be due to generalized inhibition of transcription by MG, since there were also major new peaks of RNAP binding, reflecting new promoter recognition patterns (Fig. 8C and D) and specific increases in mRNA (Fig. S4), indicating transcription of these genes. One interesting observation is the change in peak geometry as a function of increasing MG exposure. At the lowest MG concentrations, an even distribution of RNAP was observed across the TU (e.g. *xylFGHR*, *manXYZ* and *malT* in response to cAMP addition; Fig. 7C, Fig. S5A and B). However, as MG accumulated peaks became skewed towards promoter regions (Fig. 7C, Fig. S3A and B), suggesting that at high MG concentrations transcription can become paused at the promoter leading to the observed skewed peak geometry. The degree of skewing of the profiles is gene- and operon-specific indicating that the DNA sequence may itself play a role in determining the processivity of the RNAP in the presence of MG. At the time of assay that skewing becomes evident (120 min) the majority of the population is still viable (Fig. 7B). Moreover in the equivalent Type I and II experiments mRNA is still being produced (Fig. 4) and thus dead cells should not be the principal reason for the changed RNAP distribution. Guanine is the base most readily modified in the presence of MG (Krymkiewicz, 1973; Ferguson *et al*., 2000). One possibility is that the metabolism of guanine and adenine nucleotides (cAMP, ATP, GTP, ppGpp and pppGpp) has been affected, with pleiotropic consequences for RNAP activity, which would be expected to affect genes and operons differentially.

This analysis shows that *E. coli* mounts a strong transcriptional response to MG exposure, but that this may predominantly reflect the covalent modification of specific proteins and of DNA bases rather than integration of gene expression through a master regulator. Only the expression of the *kdp* genes appears to respond specifically to the activation of the protective KefGB system by MG. With the important exception of GlxI (and here only after exposure to high concentrations of MG) the genes for the protective pathways (KefGB, KefFC, GlxII, GSH biosynthesis) are not increased. This is consistent with our previous analysis that the dynamics of activation of KefGB are a critical determinant of survival (Ferguson *et al*., 1993; MacLean *et al*., 1998; Ozyamak *et al*., 2010). Although LexA/RecA is the regulatory switch for the SOS regulon, there is no precedent for these proteins being directly modulated by MG. Thus, the transcriptional changes that reflect the imbalance between intoxication, detoxification and protection, damage and repair and, a limited integration of cellular metabolism with the activation of KefGB is achieved through the formation of GSH adducts.

## Experimental procedures

### Strains and media

All experiments were performed with *E. coli* K-12 MG1655 and isogenic deletion mutants as listed in Table S1. *E. coli* K-12 strains other than MG1655 were used to create the MG1655 derivatives (see *Supporting information*). Depending on the experimental design cells were grown either in K_0.2_ minimal medium containing ∼ 0.2 mM K^+^ or K_115_ minimal medium containing ∼ 115 mM K^+^ (Epstein and Kim, 1971). Both media were supplemented with 0.2% (w/v) glucose, 0.0001% (w/v) thiamine, 0.4 mM MgSO_4_ and 6 μM (NH_4_)_2_SO_4_·FeSO_4_. In experiments conducted to stimulate MG production cells were grown in K_0.2_ medium with 0.2% (w/v) xylose as the sole carbon source and supplemented with 2 mM cAMP. Solid media contained 14 g l^−1^ agar. To prepare solid K_0.2_ medium the agar was first washed with a 1 M NaCl solution to displace trace amounts of K^+^ and then washed several times with distilled water prior to use in plates.

### Growth conditions and *in vivo* cross-linking for ChIP-chip of RNAP

Generally, overnight cultures were grown for at least 16 h at 37°C (250 rpm) and diluted into fresh pre-warmed medium to OD_650_ ∼ 0.05. Cells were grown to the relevant growth phase (see schema in Dataset S1) and cross-linked by adding 1% formaldehyde and incubation at 22°C for 20 min (70 rpm). Excess formaldehyde was quenched with 0.5 M glycine and the cells were incubated for 5 min at 22°C with shaking. Typically ∼ 1^10^ cells were harvested by centrifugation at 4°C, washed three times with ice-cold Tris-buffered-saline (pH 7.5) and cell pellets frozen at −20°C.

Cells in experiments investigating the RNAP redistribution were grown in K_0.2_ medium under three growth regimes (Type I – III; see schema in Dataset S1). For Type I experiments two parallel cultures (test and control) were inoculated (initial OD_650_ = 0.05) from a single overnight culture and grown to OD_650_ ∼ 0.4. The test culture was treated with 0.8 mM MG and both cultures were incubated further for 30 min before cross-linking. For Type II experiments a pre-culture was similarly grown to OD_650_ ∼ 0.4 and cells were diluted 10-fold into pre-warmed fresh media in the absence or presence of 0.8 mM MG and then were cross-linked after 2.5, 10 and 30 min. For Type II experiments each time point involved the sacrifice of a complete flask of culture, thus parallel flasks, each derived from the original inoculum, were used and sacrificed at different times. In addition, we conducted control experiments to assess changes in RNAP distribution solely due to dilution of cells, by comparing changes in the diluted cells to those in the pre-culture. We did not observe significant RNAP occupancy changes in these experiments (Dataset S1). Finally, to assess the potential impact of MG-induced DNA fragmentation on ChIP-chip experiments a series of controls were performed to investigate the recovery of DNA from MG-treated cells (see *Supporting information*).

Type III experiments involved the growth of cells in K_0.2_ medium with 0.2 % (w/v) xylose a carbon source. An overnight culture (with xylose) was grown for at least 24 h to allow the cells to adapt to the carbon source. A culture was grown to OD_650_ ∼ 0.4 and a defined volume containing 6 × 1^10^ cells was withdrawn to provide reference samples and cross-linked. The remainder of the culture was diluted 8-fold into pre-warmed fresh media in the presence of 2 mM cAMP (test) and cells were cross-linked after 10, 30, 60, 120 and 240 min. In addition, cells were diluted into a control culture without cAMP and cells were cross-linked upon reaching OD_650_ ∼ 0.15 (approx. 120 min). As with the Type II experiments each time point involved the sacrifice of a complete flask of culture and thus the data for different time points are derived from parallel cultures generated from a single inoculum. Subsequent ChIP-chip analysis of the cells collected at the different intervals compared changes to the reference samples from mid-exponential phase. All experiments have been replicated at least two times for ChIP-chip and independently replicated for mRNA pool determinations and assays of enzyme activities.

In experiments comparing the RNAP occupancy in cells grown in K_0.2_ and K_115_ media cultures were grown in the respective media overnight, diluted into fresh medium, grown from OD_650_ ∼ 0.05 to OD_650_ ∼ 0.4, and the cells cross-linked.

### ChIP-chip procedure

Immunoprecipitation was carried out following the procedure described by Grainger *et al*. (2004), with a modification to the lysozyme-driven cell lysis protocol. Lysozyme was used at a final concentration of 1 mg ml^−1^ (L6876, Sigma) because we observed considerable variation in the efficiency of cell lysis (30–100%) when a final concentration of 10 mg ml^−1^ was used. The lysates were sonicated 12 times for 15 s each (1 min rest) in an ice bath to shear the chromatin complexes using a Misonix sonicator 3000 (output level 4). The sonication procedure resulted in a DNA fragment range of 300–1100 bp. ChIP experiments were performed using a mouse monoclonal antibody against the β subunit of RNAP (W0002; Neoclone). Immunoprecipitated DNA samples were purified, but no amplification step was performed. Samples were processed by OGT (Oxford, UK) to incorporate Cy3 or Cy5 dyes and hybridized onto OGT 4x44K high-density oligonucleotide arrays. Routinely, control samples were labelled with Cy3 and test samples were labelled with Cy5.

### Data analysis

Data were normalized and transformed as detailed in *Supporting information*. We employed a combination of freely available data visualization and data analysis tools to detect and report peaks and supplemented the analysis with our newly developed software tool CamiScan to annotate reported peaks, enabling us to analyse large data sets more rapidly. For a more detailed description of data normalization and analysis see *Supporting information*.

### qRT-PCR

Cells were grown and treated exactly as for the ChIP experiments and RNA molecules stabilized by treating cells with RNAprotect Bacteria Reagent (Qiagen). RNA was extracted using the RNAeasy Kit (Qiagen) and reverse transcribed using the First-Strand cDNA Synthesis Kit (GE Healthcare). cDNA was quantified with a LightCycler 480 using SYBR Green (Roche). For a list of primers and a more detailed description of data normalization and analysis see *Supporting information* and Table S4.

### Cell viability and MG production assays

Assays were performed as previously described (Ozyamak *et al*., 2010), except that cells were recovered on K_0.2_ solid media for viability assays. The sensitivity of strains to MG was assessed using an MG disc assay as described in *Supporting information*.
