# Supplementary material for: Integrated stress response of Escherichia coli to methylglyoxal: transcriptional readthrough from the nemRA operon enhances protection through increased expression of glyoxalase I
Source: Mol Microbiol. 2013 May 5;88(5):936–50. doi: 10.1111/mmi.12234 (PMC3739934; doi:10.1111/mmi.12234)
Supplement: Supplementary file 1 [file mmi0088-0936-SD1.zip › mmi_12234_Supporting_Information_MMI-2013-13177.pdf]

SUPPORTING INFORMATION

Supporting Results

ChIP-chip profiles and mRNA stability/length upon MG exposure in the absence of K^+ efflux systems. DNA fragmentation on long-term exposure to MG (Ferguson *et al.*, 2000) and this has the potential to affect the accuracy of ChIP-chip analysis. The overall transcriptional response of an isogenic strain lacking both electrophile-activated K^+ efflux systems (strain MJF632) was similar to wild type strain MG1655 (SI Table 2). Typical profiles of highly transcribed genes are shown in Fig. S6. For *recN* (Fig. S6*A*) (and *recAX* and *frmRAB*; Fig. S6*D,F*) ChIP-chip profiles were unchanged and the RNAP distribution was essentially identical across >20 data points; thus the signals are not modulated when acidification of the cytoplasm is prevented. To verify that the observed RNAP distribution was not skewed by DNA damage leading to loss of DNA during extraction, the quantity of immunoprecipitated DNA (IP-DNA) was determined. Short sequences were amplified by qPCR, selecting regions from the promoter region and a section close to the middle of the *recN* gene for analysis (probes 1 & 2; Fig. S6*B*) (similar experiments were conducted with the *recAX* and *frmRAB* operons; Fig. S6*E,G*). The promoter region was always more highly represented in the IP-DNA than the selected downstream, transcribed region, as expected for the RNAP-specific antibodies. However, there was no significant difference between the wild type (MG1655) and the mutant (MJF632). To investigate whether the ChIP-chip data reflected accurately the transcription, mRNA pools were extracted from both wild type and mutant and four short segments (250 bp; probes 3-6; Fig S6*C*) were amplified by qRT-PCR. The level of mRNA detected by this protocol was found to reflect the ChIP-chip pattern, i.e. the highest levels of mRNA correspond to the 5' end of the gene and the lowest to the 3' end (Fig. S6*C*). The mRNA pools measured in this way were always lower in the mutant, but the difference between wild type and mutant was not large.

Supporting Experimental Procedures

Strains. SI Table 1 lists *E. coli* K-12 MG1655 derivatives used in experiments for which data have been presented in this study. In addition the table also lists strains that have been used to create MG1655 derivatives.

Primers. All primer sequences used in this study are listed in SI Table 4.

Creation of MG1655 derivative strain MJF632 (Δ*kefFC,* Δ*kefGB*). Briefly, an available *kefFC* null mutation in strain MJF362 was transduced into MG1655 by P1 transduction creating strain MJF631 (MG1655 Δ*kefFC::kan*). In parallel, the *kefGB* operon was deleted by a recombineering approach (Yu *et al.*, 2000) using strain DY330 (see below) and the deletion transferred into strain MJF631 by P1 transduction creating strain MJF632 (MG1655 Δ*kefFC::kan,* Δ*kefGB*<>*apr*). P1 transductions and deletion of the *kefGB* operon by recombineering were performed essentially as in (Ozyamak *et al.*, 2010). Recombineering primers (*kefGB*-KO-F, *kefGB*-KO-R) contained 50 bp homologues sequence to the 3' region of *kefB* or 50 bp homologues sequence to the region flanking *kefB* followed by 20 bp of the apramycine resistance cassette. The primers carried *HindIII* or *BstBI* restriction sites respectively for identification and selection. The recombineering strategy avoided the deletion of a potential promoter sequence for *yheV* encoded downstream of *kefB*. A possible promoter for *yheV* was found in the 3' region of *kefB* using promoter prediction software BPROM (softberry.com), thus primer *kefGB*-KO-F was designed in such a way that a short 3' region of *kefB* (189 bp) was left in place. To ensure that this region is not expressed two stop codons were incorporated into primer *kefGB*-KO-F. The deletion was verified by PCR and sequencing with primers flanking the recombination sites and primers within the apramycine cassette. Strain MJF632 did not exhibit a significantly different growth rate than the wild type strain [MJF632, $\mu = 0.72 \pm 0.01$ (n = 4); MG1655, $\mu = 0.71 \pm 0.02$ (n = 4)].

Creation of MG1655 derivative strain MJF637 (Δ*gloA*). Transfer of the *gloA* null mutation in strain MJF388 into MG1655 was achieved by P1 transduction as described above, thus creating strain MJF637.

Creation of MG1655 derivative strains lacking *frmA*, *yqhD*, *nemR* or *nemA*. In order to create MG1655 derivative mutants, we have made use of the *E. coli* gene deletion library (Keio collection, (Baba *et al.*, 2006)). This library comprises single-gene deletions of nonessential genes in *E. coli* BW25113. Mutants in this library have been created using a λ-red recombination system creating precisely-defined single-gene deletions where ORFs were replaced with a kanamycin cassette. This cassette is then used to transfer the mutation into the desired *E. coli* background strain with the option of excising the cassette using the FRT sites (FLP recognition target sites). We obtained Keio collection mutants for *frmA* (strain JW0347), *yqhD* (strain JW2978), *nemR* (strain JW5874) and *nemA* (strain JW1642). The mutation and the orientation of the kanamycin cassette was verified by PCR using primer sequences homologues to regions flanking the respective genes and primer sequences homologues to regions within the kanamycin cassette. Transfer of the gene deletions into MG1655 was achieved by P1 transduction. Mutants in the Keio collection are initially considered nonpolar because genes downstream of the mutation can be expressed from a promoter within the kanamycin cassette Kan^R (3). For our investigations we have excised the kanamycin cassette out of strains MJF633 (Δ*nemR*) and MJF634 (Δ*nemA*). Herefore, both strains were transformed with plasmid pCP20 (containing *flp)* (Cherepanov & Wackernagel, 1995) and plated onto LB plates containing ampicillin and incubated at 30˚C overnight. Single colonies were purified on LB plates containing ampicillin. Overnight cultures were grown in LB medium containing ampicillin at 30˚C and cell cultures were diluted 1:100 into fresh LB medium and grown to OD_{650nm} ~0.1 at 30˚C. Cultures were transferred to 42˚C incubated further until they reached OD_{650nm} ~0.6 since pCP20 is temperature sensitive for replication. Cells were serially diluted and plated onto LB plates and incubated overnight at 37˚C. Single colonies were chosen at random and replica plated onto LB, LB-kan and LB-amp plates to screen for colonies that were sensitive to both kanamycin and ampicillin. The respective *nemR* and *nemA* null mutants were named MJF643 and MJF644.

MG disc assays. Cells were grown overnight in K_{115} medium and 50 µl of the cell culture was spread evenly onto solid K_{115} medium. Filter discs were placed onto K_{115} plates and 5 µl of each solution to be tested was spotted onto the discs and plates were incubated for 16 h at 37°C. MG concentrations indicated are the concentrations in the solution applied and not final concentrations. Circular zones of inhibitions around the filter disks were measured and the average zone diameter across two directions was calculated and the disc diameter was subtracted from this.

GlxI enzyme assays. Cells were grown to OD_{650nm} ~0.4 in $K_{0.2}$ medium, harvested, cytoplasmic cell extracts prepared and (Ozyamak et al., 2010). GlxI activity is determined by measuring the formation of SLG spectrophotometrically at 240 nm (Racker, 1951). Enzyme assays were performed in 50 mM potassium phosphate buffer (pH 6.6) at 37°C. The reaction mixture contained 1.1 mM HTA in a final volume of 1 ml formed by the mixing 34.6 mM MG and 1.2 mM GSH (K_D of 3.1 mM was assumed for HTA formation). Enzyme activity is expressed as $U \cdot mg^{-1}$ cytoplasmic cell protein using a molar extinction coefficient of 2860 $M^{-1} \cdot cm^{-1}$ (Thornalley, 1988) and 1 unit is defined as the amount of enzyme catalyzing the formation of 1 $\mu mol \cdot min^{-1}$ SLG.

qRT-PCR. RNA molecules were stabilized by adding RNAprotect Bacteria Reagent (Qiagen) directly to cell cultures (in Type I experiments) or to cells that were rapidly filtered (in Type III experiments; cellulose acetate, 0.2 µm pore size, Whatman). RNA samples were purified using the RNAeasy Mini Kit (Qiagen). Total RNA samples usually contained small amounts of contaminating genomic DNA which was degraded using the DNA-free Kit (Ambion). RNA samples were considered to be free of genomic DNA contamination when no PCR product could be detected after 40 cycles using a primer pair to amplify the multicopy gene *insF*. Total RNA samples were assessed for degradation by assessing for the presence of sharp and distinct rRNA bands in 1.3% agarose gels. If no degradation was evident, the RNA samples were quantified using a NanoDrop system. RNA samples were stored at -80°C and defrosted on ice prior to cDNA synthesis. Synthesis of cDNA was performed using the First-Strand cDNA Synthesis Kit (GE Healthcare) with random-hexamer primers. Obtained cDNA was ready for second strand synthesis by qRT-PCR and was stored at -20°C until further use. cDNA samples were

quantified with a LightCycler 480 System (Roche) using the LightCycler 480 SYBR Green I Master Mix (Roche). qRT-PCR primer pairs were designed to amplify a product of ~200–250 bp length roughly in the middle of each gene under investigation and three reference genes (*polA, trkA, topB*). Reference genes were chosen based on the fact that these did not show changes in RNAP occupancy in any of our ChIP-chip experiments, hence predictably no changes in mRNA levels. qPCR conditions were optimized for each primer pair using sheared genomic DNA (MG1655) fragment size 500 to 1000 bp according to the following criteria: firstly, primers had to specifically amplify only one single product without primer-dimer formation, and secondly, the primers had to amplify DNA with an efficiency of at least 95%. Data were analyzed using the LightCycler 480 software (Roche) to obtain Crossing Point (CP) cycle values by applying the Second Derivative Maximum analysis. CP values were converted to a relative quantity of cDNA based on a standard curve generated for each primer pair using serially diluted amounts of sheared genomic DNA. The highest amount of DNA used for the standard curve was 50 ng and was assigned an arbitrary unit of 100000. The relative cDNA quantity for each gene under investigation was normalized against the mean of relative cDNA quantities of the reference genes in order to account for isolation, cDNA synthesis and handling differences. Where indicated gene expression changes in test samples were reported relative to control samples expressed as fold change in log_2 scale.

ChIP-chip to compare the RNAP occupancy in *E. coli* strains MG1655 and MJF632 during steady-state growth. To compare the steady state RNAP occupancy in the two strains we followed two strategies. Firstly, a ChIP-chip experiment was performed where both strains were grown in $K_{0.2}$ medium to mid-exponential phase (OD_{650nm} ~0.4), cells cross-linked and DNA immunoprecipitated. The DNA from the mutant strain MJF632 (Δ*kefGB*, Δ*kefFC*) was labeled with the Cy5 dye, DNA from the wild type strain MG1655 was labeled with the Cy3 dye and both samples were co-hybridized as before. Secondly, we performed an *in silico* analysis by using the available data sets from previous ChIP-chip experiments that assessed the impact of MG exposure on RNAP occupancy in Type I and Type II experiments. Herefore, we used data from hybridizations of control DNA samples labeled with the Cy3 dye. For each strain the data sets of the Cy3 fluorescence intensities from replicate experiments were averaged. The averaged fluorescence intensity data from the mutant strain MJF632 were assigned to be the test condition ("Cy5"), and the data for MG1655 were considered to be the control condition ("Cy3"), thereby matching the experimental labeling of DNA in the real experiment. Data were then transformed and normalized as for data derived from wet lab experiments are above. Data can be found in SI Dataset 1.

ChIP-chip data analysis

Each ChIP-chip data set was analyzed separately and peaks and troughs, i.e. genomic areas with increased and decreased RNAP occupancy, were assessed both visually and using available software ChIPOTle (Buck *et al.*, 2005). In the framework of this study we developed a new software tool, named CamiScan, that complements the ChIPOTle tool by annotating peaks automatically and matching peak coordinates to genome annotation files, thereby significantly improving ChIP-chip data analysis and data reporting (SI Dataset 2).

ChIPOTle uses a sliding window approach and reports the locations of statistically significant peaks and was found to perform well on our data. We took advantage of its output format to develop CamiScan (SI Dataset 2), a Perl application that matches/superimposes peak information to a GenBank file containing information about genomic features (e.g. U00096.gb for *E. coli* MG1655). CamiScan states names, functions and products of the genes found under detected peaks in ChIP-chip data. This allowed us a faster assessment of RNAP binding in open reading frames and intergenic regions of the *E. coli* chromosome. A detailed description of the data analysis procedure and the CamiScan tool can be found further below.

Data Extraction. Data were collected using an Agilent Technologies G2505B scanner and extracted using the Agilent feature extraction software 9.5.3.1, using local background correction.

Data visualization. In the first instance raw data were assessed visually by loading raw data files (Agilent format; *.txt) into the freely available OGT ChIP browser (http://www.ogt.co.uk). Processed and normalized data sets were submitted into the ArrayExpress database in GFF format to also allow easy visualization of the data with the genome browser implemented in the EcoCyc database.

Microarrays, data pre-processing and normalization. We used custom-designed OGT high density oligonucleotide microarrays that comprised 43450 oligonucleotide probes (60-mers) representing the entire *E. coli* MG1655 genome (U00096) at an average gap space of ~106 bp. The array design was submitted to the ArrayExpress database under accession number A-MEXP-1557. Fluorescently labeled DNA samples were hybridized and data were extracted as described above. For each probe Cy5 and Cy3 fluorescence intensities were obtained, data (columns "rProcessedSignal" and "gProcessedSignal", respectively) and systematic probe names were copied from Agilent raw data files into MS Excel spreadsheets. The physical location of the probes on the array is random and not according to their genomic context to prevent hybridization biases. Thus, data points were sorted according to their order in the *E. coli* MG1655 genome using MS Excel operations. The order of the probes can be derived from the systematic probe names (e.g. CHiP_U00096_0000137, designed by OGT), which follow a pattern where the last 7 digits correspond to the starting base position of that probe.

We routinely assessed the overall distribution of $\log_2$ (Cy5/Cy3) ratios for raw data before further data manipulation. $\log_2$ ratios followed a near Gaussian distribution where the median deviated from 0. It is worth emphasizing that ChIP-chip assays performed in this study differ from classical ChIP-chip assays in that both DNA samples are derived from an IP, which has an effect on the signal distribution observed. This is in marked contrast to assays in which the control/reference is genomic DNA. In the latter experiments the logarithmic signal ratios will be distributed asymmetrically because of the presence of two populations of DNA fragments that are hybridized onto the array (Buck & Lieb, 2004, Scacheri *et al.*, 2006). The first population corresponds to the non IP-enriched genomic DNA and follows a random, Gaussian distribution. The second population consists of the specifically IP-enriched DNA fragments, which skews the Gaussian distribution to the right. The skewness may be dependent on the abundance of the assayed DNA-binding protein and strength of the interaction with the DNA. While ChIP-chip studies on histone proteins in eukaryotes have been reported to result in almost bimodal distributions, where noise and enrichment can be distinguished easily (Scacheri et al., 2006), the distribution might only exhibit a 'bump' in the right side of the Gaussian distribution meaning that the noise and enrichment distributions overlap heavily and thus making it difficult to distinguish real binding events that are close to the noise level. Signal ratios on the negative side of the distribution are not meaningful because there can only be enrichment of DNA, not depletion and thereby ratios on the negative side can be ignored in the analysis. In contrast, in this study noise distribution could be skewed towards both ends of the distribution and large fluorescent ratios on the negative side, as well as on the positive side of the distribution, are potentially of interest. $\log_2$ ratios in this study followed a near Gaussian distribution where the median deviated from 0. This is mainly because the Cy5 and Cy3 fluorescent dyes exhibit different extinction coefficients (ε_{Cy5} is approximately 1.66-fold higher than ε_{Cy3}). Data sets in which the same amount of Cy5- and Cy3 labeled DNA is hybridized, and assuming similar labeling efficiencies, will show a median $\log_2$ (Cy5/Cy3) above 0 (roughly 0.76). In order to work with a common median for all data sets, the median was subtracted from the $\log_2$ ratios for individual probes shifting the distribution and centering the data sets at 0. The centering of the logarithmic data around 0 is helpful in the assessment of relative enriched or depleted genomic regions in the ChIP-chip data and provides an intuitive midpoint; positive $\log_2$ ratios will indicate enrichment and negative ratios will indicate depletion of specific DNA fragments. This global normalization procedure assumes that the majority of the data points do not change significantly and there is an approximately equal number of increased and decreased RNAP binding events. This assumption may not be true in Type III experiments, especially in sampled cells that had been incubated extensively in the presence of cAMP ($t_{240\ min}$), correlating with cell death. In Type III experiments we applied a different normalization procedure for reasons discussed. We assessed the overall distribution of average fluorescence intensity values for both Cy5 and Cy3 dyes in these experiments (average was representative for the two replicate data; Cy3 values were adjusted for the lower extinction coefficient). As mentioned before, in Type III experiments DNA immunoprecipitated from cAMP-treated and reference cells were labeled with Cy5 and Cy3, respectively. Both Cy5 and Cy3 overall signals followed a near Gaussian distribution with an extended tail towards higher intensity values. This is in line with the fact that the majority of genomic regions exhibit RNAP occupancies within a "background" level, whereas others show very high intensity values, indicative of high transcriptional activity across these regions. The distribution of Cy5 signals up to and including t_{120min} were almost super imposable with an integrated absolute area of ~ 65175000 and an x_0 = ~55750 (bin center with the highest frequency) for each. However, the Cy5 signal distribution at t_{240min} was shifted towards the lower intensity values (x_0 = 21750), although the

integrated absolute area was identical to previous time points. Higher fluorescence intensities for a probe indicate a higher abundance of DNA fragments that hybridize to it. Therefore, it can be assumed that an overall shift towards lower intensity values indicates either a generally lower DNA fragment recovery or differences in labeling efficiencies between DNA recovered at t_{240min} and earlier. It is conceivable that the prolonged exposure of the cells to MG results in extensive modification of the genomic DNA and ultimately interferes with the labeling using Cy5-CTP. We have also analyzed the distribution of Cy3 signals at the different time points and these were very similar for up to and including t_{120min} (integrated absolute area = ~ 65175000; x_0 = ~ 38500). In contrast to the Cy5 signals, at t_{240min} there was a shift towards higher intensity values (x_0 = ~ 58750; integrated absolute area = ~ 65175000). Theoretically the Cy3 signal distribution across all time points should be identical since reference cells used to immunoprecipitate DNA had a common source. Immunoprecipitations were performed on the same day using same antibody solution and DNA labeling and hybridization performed on the same day. This change at t_{240min} may indicate an anomalous enhancement of Cy3 fluorescence. The photophysical properties of fluorescent probes may depend on the physical and chemical properties of the surroundings, and (Gruber *et al.*, 2000) had reported anomalous behavior of Cy3 upon covalent linking to IgG and noncovalent binding to avidin. We are currently uncertain about the molecular reasons for the behavior observed here. Due to the reasons presented above we chose to normalize ChIP-chip data from Type III experiments with respect to 8 reference genomic regions that exhibited very low signal intensities (i.e. low RNAP binding) in both cAMP-treated and untreated cells and that did not change over time. The following genes/operons were chosen across the MG1655 genome and these are mostly cryptic or prophage related genes: *phn* operon, *rhsA*, *rhsC*, *rhsD*, *rhsE*, *stfR*, *yggF*, *yagEF*. An average $\log_2$ ratio for the corresponding regions was calculated, data sets were then normalized, i.e. $\log_2$ (Cy5 / Cy3) ratios for the entire genome were shifted so that the average $\log_2$ ratio for the reference regions was 0.

Peak detection using ChIPOTle. The visual / manual data analysis of a relatively abundant DNA-binding protein such as RNAP in large data sets is a laborious process, hence, we sought to analyze the data sets in an automated fashion. The ChIPOTle algorithm (Buck et al., 2005) was used to detect peaks after pre-processing the data as described above. ChIPOTle is implemented as a user-friendly MS Excel application and requires relatively little technical expertise. ChIPOTle determines peaks by calculating the average $\log_2$ ratio for a user defined window (e.g. 1000 bp) and by sliding this window step wise along a given genome. A window size of 1000 bp and a step size of 100 bp generally performed well on our data. ChIPOTle offers three significance criteria for determining the significance of enrichment and a simple peak height cut-off criterion was chosen in this study. For each data set we have determined a cut-off value by fitting a Gaussian function to the normalized $\log_2$ (Cy5/Cy3) distribution using the software Origin 8 (OriginLab Corporation). We based subsequent significance criteria for peak detection on the standard deviation of the fitted distribution. Generally, the distribution could be described well with a Gaussian function (adjusted R^2 of 0.94 - 0.96). Based on the assumption that the majority of the data points constitute noise/background we chose the cut-off as the value in which 95% of the data are within the Gaussian distribution, i.e. a confidence interval of 95% (cut-off = σ * 1.96). In a few experiments when the noise-to-signal ratio was high, we chose a cut-off value that resulted in a reasonable good match of peaks detected on visual inspection and ChIPOTle. Peak height cut-off values for ChIPOTle are given in the SI Dataset. As pointed out above, our experimental design allows us to analyze the enrichment as well as the "depletion" of specific genomic areas, which technically speaking would be defined as negative peaks or troughs. Since ChIPOTle does report troughs, we analyzed the two sides of the $\log_2$ ratio distribution separately. To detect RNAP peaks corresponding to the enrichment of genomic fragments we have analyzed $\log_2$ (Cy5/Cy3) ratios. In order to detect genomic regions with decreased RNAP occupancy we analyzed $\log_2$ (Cy3/Cy5) ratios. For each data set, ChIPOTle was run on three overlapping subsets of data, due to a limitation of MS Excel with regard to total number of windows. Peaks in the ChIPOTle output sheet "Peaks above cutoff" were compiled into a single list and reoccurring peaks were removed manually. Peak numbers were reassigned in consecutive, ascending order and the spreadsheet saved as a tab-delimited .txt file and used as CamiScan input file. It is common to average ChIP-chip data from biological replicate experiments, however, we find that averaging can sometimes abolish strong ChIP-chip signals or may indicate occupancy events that are not supported by other replicates. Hence, normalized $\log_2$ ratios from independent replicate experiments were processed and analyzed separately. Only for Type III experiments we chose to average data sets to reduce the data complexity.

CamiScan software and peak annotation. Analysis of the Chip-Chip data was performed by using the ChIPOTle software as described above. Our new CamiScan software utilizes only the "Peaks above cutoff" output information contained in the ChIPOTle MS Excel spread sheet. This spreadsheet is composed of 8 columns and contains quantitative data such as the position and the length of peaks in relation to the genome. However, ChIPOTle does not list detailed information about the correlation of genes or gene products to a certain peak. CamiScan collects such information from an annotated GenBank file for the genome sequence of the organism which can be downloaded from a database such as NCBI (http://www.ncbi.nlm.nih.gov/Database/). CamiScan is a Perl script that is able to perform a comparison between a peak list file (tab-delimited *.txt file, see above) and a GenBank information file for the organism of interest (e.g. U00096.gb). CamiScan generates an output file containing a characterization of each peak by describing corresponding gene names, their types (e.g. tRNA, rRNA), products and functions. Also specified is the percentage of coverage for each gene. This indicates to what extend a certain gene is covered by a particular peak. For instance, peak lengths in ChIP-chip of RNAP usually cover the full length of genes or transcription units (TUs). Exceptions from this may occur depending on the experiment and biological phenomena such as stalled RNAP. For more information on the interpretation of this parameter see below. Furthermore, CamiScan can annotate peaks that are positioned between genes and highlights these as "intergenic" and stating information about genes flanking the peaks. The algorithm performs a continuous unidirectional search through the GenBank file, extracting relevant information that matches specific string patterns, guided by the peak position information contained in the peak list file. The original information contained in the peak list file is maintained in the CamiScan output file which consists of a 12-column tab-delimited .txt file, with gene name, function, product and percentage columns added to the ChIPOTle columns. In this way CamiScan complements ChIPOTle and provides an easy access to qualitative information by listing the gene names related to each peak as well as their description, facilitating the analysis and comparison of high numbers of experiments performed. CamiScan can, in principle, be applied to any type of data, i.e. data from ChIP-chip of RNAP or of any specific DNA binding protein, however, it is designed to extract information from GenBank file of a prokaryotic organism.

Running CamiScan. The CamiScan tool is enclosed in SI Dataset 2. Prior to using CamiScan, a Perl distribution must be installed on the user's machine. Most operating systems have a Perl distribution on it, but may require updating. A current version can be found online and installed easily on the user's computer (go to: http://www.activestate.com/activeperl). CamiScan is implemented as a user-friendly interface application that is launched upon double clicking (**please see note further below, ***). The user is prompted to browse for both input files (peak list and GenBank file, see above) (Fig. S7) and then to press the "Run" button. Upon successful running, CamiScan creates an output file named after the original peak file by adding "_output" to its original name (e.g. "filename_output.txt") and this file will be placed in the same location as the input file. The output file can easily be read with MS Excel or any other application that can open tab-delimited txt files.

***** *Due to the Molecular Microbiology file submission policy we could not provide CamiScan in its original file format (CamiScan.pl). The file in SI Dataset 2 is a .txt file. To launch CamiScan, please right-click and choose to open the file with the "Perl Command Line Interpreter", an option available once activeperl is installed on your computer. Alternatively, please contact corresponding authors to request the application.*

Interpretation of CamiScan output files and peak calling. CamiScan compiles important information from a GenBank file that facilitates an improved analysis of the ChIP-chip experiments. However, one has to bear in mind that CamiScan is only guided by ChIPOTle software. In ChIP-chip experiments the abundance of a random population of DNA fragments of various lengths is determined. The precise boundaries of these fragments are not determined by the position of the RNAP, but the enrichment by IP is dependent on RNAP bound to the DNA. ChIPOTle cannot detect gene boundaries with 100% accuracy due to the random shearing of the DNA. The consequence is that genomic areas at both sides of the actual binding site for RNAP will be enriched despite the absence of RNAP from those adjacent genes. CamiScan does not take this effect into consideration and will also report those genomic areas to be enriched. The reported flanking regions may represent genuine enrichment (i.e. transcription) and must be confirmed experimentally. Occasionally the RNAP occupancies extend beyond known TUs and these data may be the first indication of larger TUs than previously reported. In

addition, detected peaks may not be symmetrical across the known TU, a phenomenon that may have many sources, including the failure of transcription elongation at one or more gene boundaries in an operon. However, also difficulties with the IP should be considered. A visual assessment and interpretation of the ChIP-chip data is especially recommended in these cases.

References:

Baba, T., T. Ara, M. Hasegawa, Y. Takai, Y. Okumura, M. Baba, K. A. Datsenko, M. Tomita, B. L. Wanner & H. Mori, (2006) Construction of *Escherichia coli* K-12 in-frame, single-gene knockout mutants: the Keio collection. *Mol Syst Biol* **2**: 2006 0008.

Buck, M. J. & J. D. Lieb, (2004) ChIP-chip: considerations for the design, analysis, and application of genome-wide chromatin immunoprecipitation experiments. *Genomics* **83**: 349-360.

Buck, M. J., A. B. Nobel & J. D. Lieb, (2005) ChIPOTle: a user-friendly tool for the analysis of ChIP-chip data. *Genome Biol* **6**: R97.

Cherepanov, P. P. & W. Wackernagel, (1995) Gene disruption in *Escherichia coli*: TcR and KmR cassettes with the option of Flp-catalyzed excision of the antibiotic-resistance determinant. *Gene* **158**: 9-14.

Ferguson, G. P., J. R. Battista, A. T. Lee & I. R. Booth, (2000) Protection of the DNA during the exposure of *Escherichia coli* cells to a toxic metabolite: the role of the KefB and KefC potassium channels. *Mol Microbiol* **35**: 113-122.

Gruber, H. J., C. D. Hahn, G. Kada, C. K. Riener, G. S. Harms, W. Ahrer, T. G. Dax & H. G. Knaus, (2000) Anomalous fluorescence enhancement of Cy3 and cy3.5 versus anomalous fluorescence loss of Cy5 and Cy7 upon covalent linking to IgG and noncovalent binding to avidin. *Bioconjug Chem* **11**: 696-704.

Ozyamak, E., S. S. Black, C. A. Walker, M. J. Maclean, W. Bartlett, S. Miller & I. R. Booth, (2010) The critical role of *S*-lactoylglutathione formation during methylglyoxal detoxification in *Escherichia coli*. *Mol Microbiol* **78**: 1577-1590.

Racker, E., (1951) The mechanism of action of glyoxalase. *J Biol Chem* **190**: 685-696.

Scacheri, P. C., G. E. Crawford & S. Davis, (2006) Statistics for ChIP-chip and DNase hypersensitivity experiments on NimbleGen arrays. *Methods Enzymol* **411**: 270-282.

Thornalley, P. J., (1988) Modification of the glyoxalase system in human red blood cells by glucose in vitro. *Biochem J* **254**: 751-755.

Yu, D., H. M. Ellis, E. C. Lee, N. A. Jenkins, N. G. Copeland & D. L. Court, (2000) An efficient recombination system for chromosome engineering in *Escherichia coli*. *Proc Natl Acad Sci U S A* **97**: 5978-5983.

Legends of SI Figures, SI Tables and SI Datasets.

Fig. S1. Chromosomal organization of the *nemRA* operon and the *gloA* gene. Experimental and predicted promoters are shown.

Fig. S2. Transcriptional response to lethal MG concentration (Type II experiment) is rapid; LexA-regulated (*A*, *B*) *and* OxyR-regulated genes (*C-F)* exhibit different temporal expression patterns. Two independent experiments were performed for each time point; averaged and smoothed data sets are shown. Data smoothing and labels are as described in Fig. 3.

Fig. S3. RNAP occupancy across selected genes upon progressive MG accumulation in *E. coli* MG1655. RNAP occupancy across CRP-regulated genes (*A*, *B*), the *frmRAB* operon and *yqhD-dkgA* genes at different time points after diluting cells into $K_{0.2}$ medium containing cAMP, relative to reference cells (Type III experiments). Data set, smoothing and labels are as described in Fig. 3.

Fig. S4. Transcriptional changes for selected genes upon progressive MG accumulation in *E. coli* MG1655 as determined by qRT-PCR. MG1655 cells were grown as detailed for Type III experiments and cells were sampled at the same time points as in ChIP-chip experiments. Total RNA was stabilized, isolated and reverse transcribed into cDNA from reference, control and test cells at different

time points. Transcript levels in each sample were normalized against the internal control genes *topB*, *trkA* and *polA*. Changes in transcript levels are expressed as fold-changes relative to reference samples. Error bars indicate the standard deviation of three independent experiments.

Fig. S5. RNAP occupancy across selected the *kdp* and *nemRA* operons genes upon progressive MG accumulation in *E. coli* MG1655. RNAP occupancy across *kdp* operons (*A*) and *nemRA* (*B*) at different time points after diluting cells into $K_{0.2}$ medium containing cAMP, relative to reference cells (Type III experiments). Data set, smoothing and labels are as described in Fig. 3.

Fig. S6. RNAP occupancy and transcript length is not effected in cells lacking the MG-protective K^+ efflux systems KefB and KefC. RNAP occupancy across *recN* (*A*), *recAX* (*D*) and *frmAB* (*F*) upon MG stress in strain MG1655 (filled diamonds) and strain MJF632 (Δ*kefGB*, Δ*kefFC*; open diamonds). Data from representative Type I experiments are shown. Data smoothing and labels are as in Fig. 3. Numbered black bars indicate areas primed for qPCR experiments on immunoprecipitated DNA (*B*, *E*, *G*) and and cDNA (*C*). (*E, G*) Black bars: MG1655, open bars: MJF632. The mean and standard deviation of three independent experiments are shown. (*C*) Data are representative of two independent replicates. Both IP-DNA and cDNA were derived from cell populations separate to those used for ChIP-chip.

Fig. S7. User interface of the CamiScan software. See please see text above for more information and detailed user description.

SI Table 1. *E. coli* strains used in this study.

SI Table 2. Genomic regions of *E. coli* MG1655 and MJF632 (Δ*kefGB*, Δ*kefFC*) that exhibit the highest RNAP occupancies in Type I and Type II experiments. Replicate data sets for each strain were averaged and then analyzed using ChIPOTle and CamiScan to reduce the data complexity. RNAP occupancy was also assessed visually. For a complete list see SI Dataset files (CamiScan_Output_Data_Files). $\log_2$ ratios are “High Average Ratios” as calculated by the ChIPOTle software. LexA-regulated genes are highlighted in bold.

SI Table 3. Genomic regions of *E. coli* MG1655 that exhibit the highest RNAP occupancies in Type III experiments. ChIP-chip signals for test DNA (cAMP-treated) at t_{30min}, t_{60min}, t_{120min} and t_{240min} was compared *in silico* to test DNA signals at t_{10min}, thus visually eliminating cAMP-induced changes and highlighting MG induced changes in RNAP occupancy. Data sets (same as used in Fig. 6) were averaged, and then analyzed using ChIPOTle and CamiScan. RNAP occupancy was also assessed visually. $\log_2$ ratios are “High Average Ratios” as calculated by the ChIPOTle software. LexA-regulated genes are highlighted in bold.

SI Table 4. List and sequence of primers used in this study.

SI Dataset 1. List of all ChIP-chip experiments, schema for experimental design of Type I-III experiments and CamiScan output files in MS Excel worksheet format.

SI Dataset 2. CamiScan software - please see text above for more information.

Fig. S1

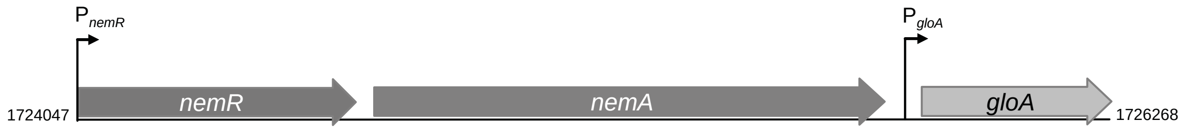

P_{nemR} : experimental evidence

P_{gloA} : bioinformatic analysis (RegulonDB)

Fig. S2

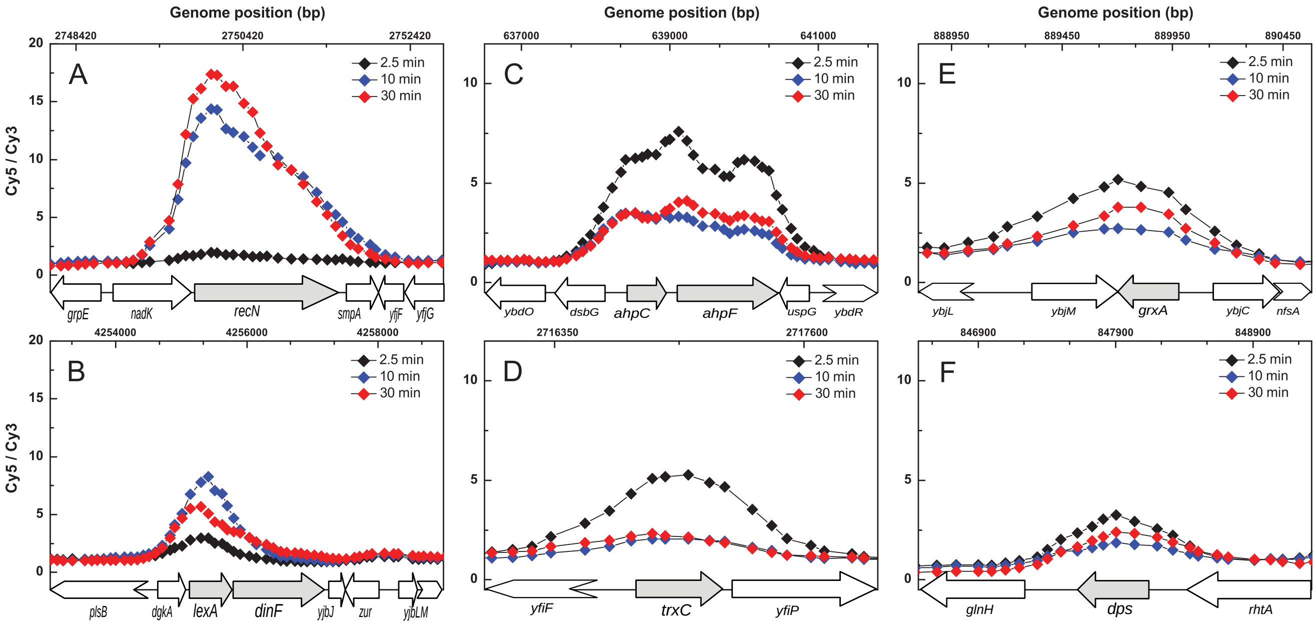

Fig. S3

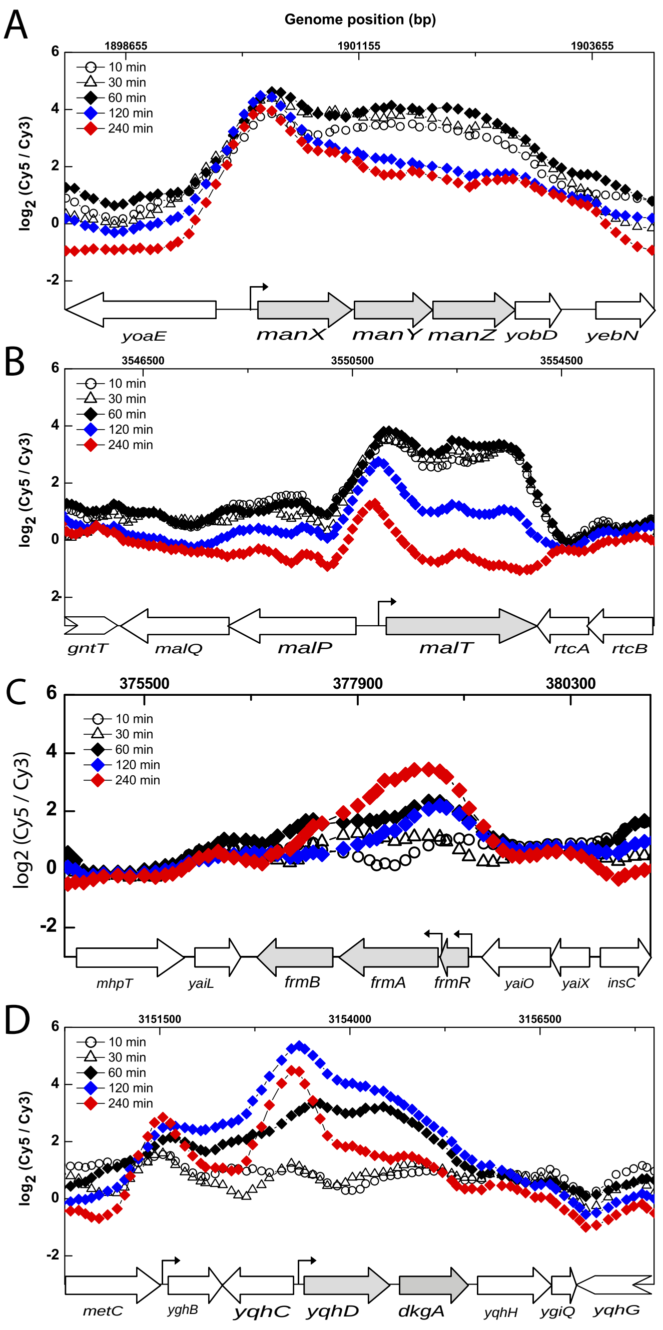
A
Genome position (bp)
1898655
1901155
1903655
log2 (Cy5 / Cy3)
10 min
30 min
60 min
120 min
240 min
yoaE
manX
manY
manZ
yobD
yebN
B
3546500
3550500
3554500
gntT
malQ
malP
malT
rtcA
rtcB
C
375500
377900
380300
log2 (Cy5 / Cy3)
mhpT
yaiL
frmB
frmA
frmR
yaiO
yaiX
insC
D
3151500
3154000
3156500
metC
yghB
yqhC
yqhD
dkgA
yqhH
ygiQ
yqhG

Fig. S4

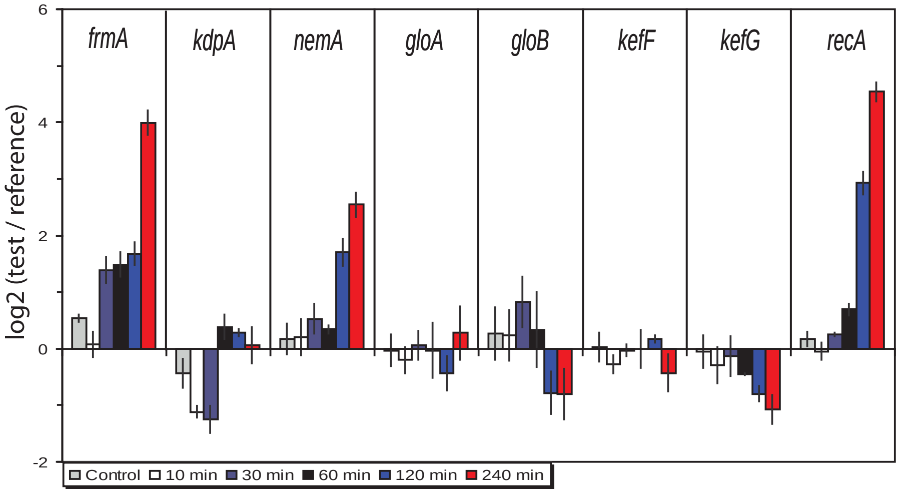

frmA
kdpA
nemA
gloA
gloB
kefF
kefG
recA
log2 (test / reference)
6
4
2
0
-2
Control
10 min
30 min
60 min
120 min
240 min

Fig. S5

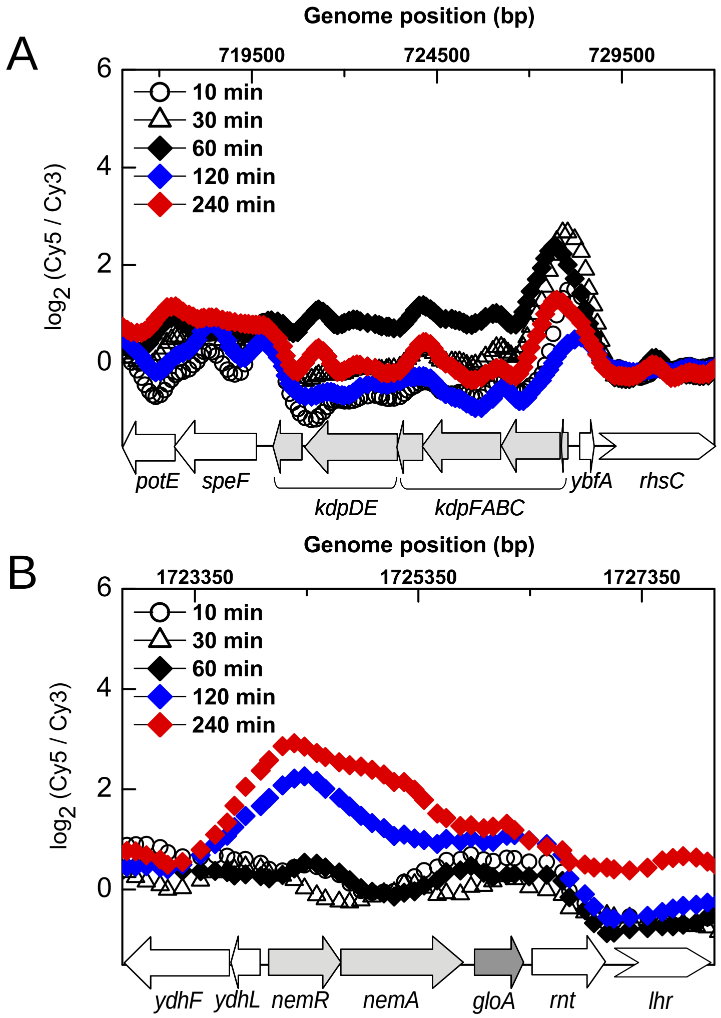

A
Genome position (bp)
719500
724500
729500
6
4
2
0
log2 (Cy5 / Cy3)
10 min
30 min
60 min
120 min
240 min
potE
speF
kdpDE
kdpFABC
ybfA
rhsC
B
Genome position (bp)
1723350
1725350
1727350
6
4
2
0
log2 (Cy5 / Cy3)
10 min
30 min
60 min
120 min
240 min
ydhF
ydhL
nemR
nemA
gloA
rnt
lhr

Fig. S6

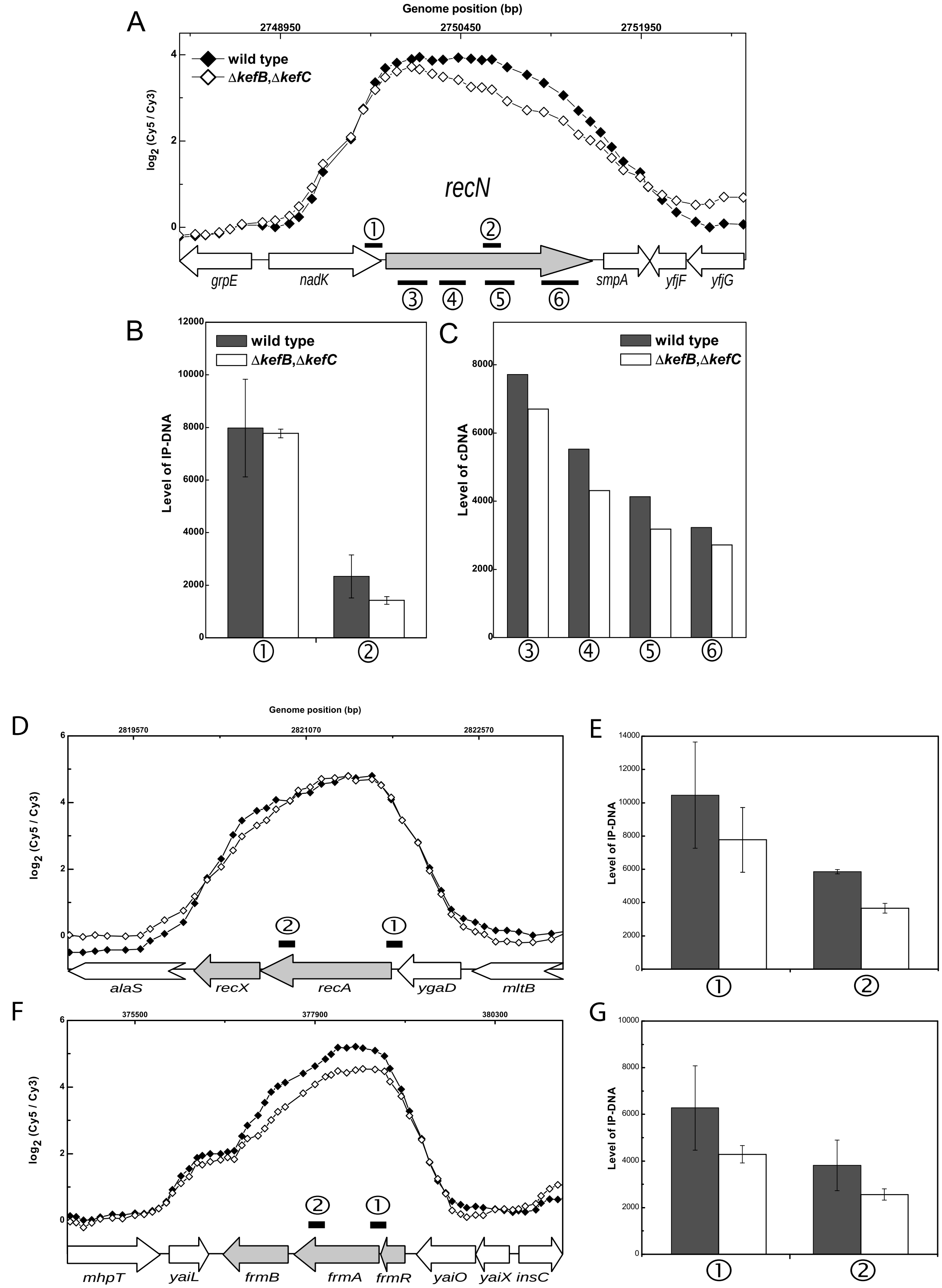
A
Genome position (bp)
2748950
2750450
2751950
wild type
ΔkefB,ΔkefC
log2 (Cy5 / Cy3)
recN
grpE
nadK
smpA
yfjF
yfjG
B
Level of IP-DNA
C
Level of cDNA
D
Genome position (bp)
2819570
2821070
2822570
alaS
recX
recA
ygaD
mltB
E
F
375500
377900
380300
mhpT
yaiL
frmB
frmA
frmR
yaiO
yaiX
insC
G

Fig. S7

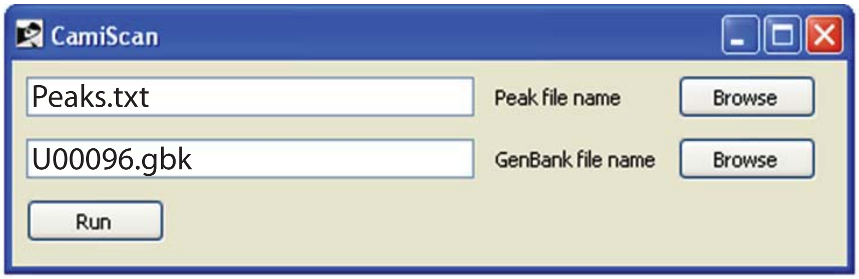
CamiScan
Peaks.txt
Peak file name
Browse
U00096.gbk
GenBank file name
Browse
Run

SI Table 1

Strain	Genotype	Source / Reference
MG1655	*F- λ- ilvG rfb50 rpH1*	(Blattner *et al.*, 1997)
MG1655 Δ*kdpA*	MG1655 *rpsL150 kdpA4::rspL-neo-kan*	(Heermann *et al.*, 2008)
DY330	W3110 Δ*lacU169 gal490* λ*cI857* Δ*(cro-bioA)*	(Yu *et al.*, 2000)
MJF362	Frag5 Δ*kefFC::kan*	I.R. Booth
MJF631	MG1655 Δ*kefFC::kan*	this study
MJF632	MG1655 Δ*kefFC::kan,* Δ*kefGB<>apr*	this study
JW5874	BW25113 Δ*nemR::kan*	(Baba *et al.*, 2006)
JW1642	BW25113 Δ*nemA::kan*	(Baba et al., 2006)
JW0347	BW25113 Δ*frmA::kan*	(Baba et al., 2006)
JW2978	BW25113 Δ*yqhD::kan*	(Baba et al., 2006)
MJF635	MG1655 Δ*frmA::kan*	this study
MJF636	MG1655 Δ*yqhD::kan*	this study
MJF633	MG1655 Δ*nemR::kan*	this study
MJF634	MG1655 Δ*nemA::kan*	this study
MJF643	MG1655 Δ*nemR*	this study
MJF644	MG1655 Δ*nemA*	this study
MJF388	MJF274 Δ*gloA::kan*	(MacLean *et al.*, 1998)
MJF637	MG1655 Δ*gloA::kan*	this study

References:

Baba, T., T. Ara, M. Hasegawa, Y. Takai, Y. Okumura, M. Baba, K. A. Datsenko, M. Tomita, B. L. Wanner & H. Mori, (2006) Construction of Escherichia coli K-12 in-frame, single-gene knockout mutants: the Keio collection. *Mol Syst Biol* **2**: 2006 0008.

Blattner, F. R., G. Plunkett, 3rd, C. A. Bloch, N. T. Perna, V. Burland, M. Riley, J. Collado-Vides, J. D. Glasner, C. K. Rode, G. F. Mayhew, J. Gregor, N. W. Davis, H. A. Kirkpatrick, M. A. Goeden, D. J. Rose, B. Mau & Y. Shao, (1997) The complete genome sequence of Escherichia coli K-12. *Science* **277**: 1453-1462.

Heermann, R., T. Zeppenfeld & K. Jung, (2008) Simple generation of site-directed point mutations in the Escherichia coli chromosome using Red(R)/ET(R) Recombination. *Microb Cell Fact* **7**: 14.

MacLean, M. J., L. S. Ness, G. P. Ferguson & I. R. Booth, (1998) The role of glyoxalase I in the detoxification of methylglyoxal and in the activation of the KefB K+ efflux system in Escherichia coli. *Mol Microbiol* **27**: 563-571.

Yu, D., H. M. Ellis, E. C. Lee, N. A. Jenkins, N. G. Copeland & D. L. Court, (2000) An efficient recombination system for chromosome engineering in Escherichia coli. *Proc Natl Acad Sci U S A* **97**: 5978-5983.

SI Table 2

MG1655, Type I exp., 30 min		MJF632, Type I exp., 30 min		MG1655, Type II exp., 30 min		MJF632, Type II exp., 30 min	
Genes	$\log_2$ (Cy5/Cy3)	Genes	$\log_2$ (Cy5/Cy3)	Genes	$\log_2$ (Cy5/Cy3)	Genes	$\log_2$ (Cy5/Cy3)
frmRAB	2.46	***recAX***	2.77	***recAX***	4.43	***recAX***	4.80
yebG	2.18	***recN***	2.47	***yebG***	4.06	*frmRAB*	4.48
recAX	2.16	*frmRAB*	2.19	***recN***	3.95	***yebG***	4.23
recN	1.87	***yebG***	2.08	*frmRAB*	3.75	***recN***	3.93
sulA	1.32	*deaD*	1.63	***sulA***	3.43	***sulA***	3.32
lexA-dinF	1.28	***sulA***	1.56	*yqhD-dkgA*	3.40	*yqhD-dkgA*	3.13
uvrA	1.21	***uvrA***	1.52	***umuCD***	2.55	*sbp*	2.93
rRNA operon rrsH	1.16	***lexA-dinF***	1.49	***uvrA***	2.40	***lexA-dinF***	2.84
plaP	1.14	***tisB***	1.47	*kdpFABCDE*	2.39	***uvrA***	2.53
dinI	1.14	*rpsJ to rplP*	1.44	*cusFBA*	2.32	*cusFBA*	2.49
rRNA operon rrsE	1.11	***dinI***	1.39	*alaE*	2.22	*rpsJ to rplP*	2.47
glyVXY	1.11	*yqhD-dkgA*	1.39	***lexA-dinF***	2.16	*deaD*	2.44
yqhD-dkgA	1.11	*cysJ*	1.21	*deaD*	2.07	*gcvA / gcvB*	2.44
rRNA operon rrsC	1.09	*rpsLG-fusA-tufA*	1.19	*rpsJ to rplP*	2.02	*iscRSUA*	2.36
rRNA operon rrsB	1.08	*rsxABCD*	1.18	***tisB***	2.00	*rpsU - dnaG*	2.13
rRNA operon rrsA	1.06	*thrU to-tufB / rplJL*	1.17	*iscRSUA*	1.89	*nemA / gloA*	2.12
umuCD	1.05	*rplKAJL*	1.17	*ahpFC*	1.84	*marRAB*	2.09
rRNA operon rrsF	1.05	*phn*	1.17	***dinB***	1.76	***tisB***	2.08
lysT-valT-lysW	1.03	*rpsU - dnaG*	1.16	*nemA / gloA*	1.76	***umuCD***	1.97
kdpFABCDE	1.02	*nemA / gloA*	1.14	***dinI***	1.74	*ahpFC*	1.97
rrsG-gltW-rrlG-rrfG	1.00	*cysK*	1.10	*sbp*	1.68	*thrU to-tufB / rplJL*	1.91
ydjM	0.99	*sbp*	1.09	*rplU-rpmA / yhbE-obgE*	1.66	*cysK*	1.86
glnW-metU-glnVX	0.97	*pdhR - aceE*	1.08	*gcvA / gcvB*	1.65	*rplU-rpmA / yhbE-obgE*	1.80
infC-rpmI-rplT	0.96	*gabDT*	1.08	***uvrB***	1.60	***uvrB***	1.78
rpmG	0.90	*glyVXY*	1.07	*grxA*	1.57	*rRNA operon rrsB*	1.72
rsxABCD	0.90	***uvrB***	1.02	*yahA*	1.35	*rRNA operon rrsE*	1.69
yahA	0.89	*rhsB / nikE*	1.02	*yeaJ*	1.33	*rRNA operon rrsC*	1.69
ruvAB	0.87	*yceD*	1.00	*dcyD - yecS*	1.31	***ydjM***	1.68
rpsT	0.87	*dnaX*	1.00	*rpsU - dnaG*	1.30	***dinI***	1.67
deaD	0.85	*dnaA*	1.00	***dinD***	1.29	*rpsP-rimM-trmD-rplS*	1.66
yceD	0.85	*rRNA operon rrsE*	0.98	*yjiC / iraD*	1.25	*yceD / fabF*	1.65
fabF	0.85	*rRNA operon rrsB*	0.96	*rimP-nusA-infB*	1.24	*plaP*	1.65
pdhR-aceE	0.84	*iscRSUA*	0.95	***ruvAB***	1.23	*rRNA operon rrsH*	1.65
thrU to-tufB / rplJL	0.83	***ruvAB***	0.95	*yegQ*	1.22	*insAB*	1.60
rpsU - dnaG	0.82	*plaP*	0.95	*plaP*	1.22	***dinB***	1.58
uvrB	0.81	***umuCD***	0.94	*rpsP-rimM-trmD-rplS*	1.20	*grxA*	1.57
isrB	0.81	*rhsC*	0.92	*cspB*	1.19	*rRNA operon rrsA*	1.56
rpsB- tsf	0.81	*rplU-rpmA / yhbE-obgE*	0.92	*marRAB*	1.18	*ycfQ / bhsA*	1.51
rpsR-rplI	0.81	*infC-rpmI-rplT*	0.92	***ydjM***	1.13	*dusB / fis*	1.50
waaJ / waaY	0.80	*waaJ / waaY*	0.91	***dinG***	1.10	*yciW*	1.49
ydfO	0.79	*rpsP-rimM-trmD-rplS*	0.91	***uvrD***	1.08	*alaE*	1.48
yaiS	0.77	*rhsA-yibA*	0.91	*yohJK*	1.03	*rpsR - rplI*	1.46
rpsP-rimM-trmD-rplS	0.75	***ydjM***	0.90	*cysJ*	1.02	*ygeG*	1.44
cspA / hokA	0.74	*rRNA operon rrsC*	0.89	*cysK*	1.01	*rrsG-gltW-rrlG-rrfG*	1.44
ydcD	0.73	*lpxC*	0.89	*polB*	1.00	*cysJ*	1.42
rpsJ to rplP	0.71	*insEF-1*	0.89	*trxC*	1.00	*rnlAB*	1.41
yddK	0.71	*gcvA / gcvB*	0.87	*rlmN*	0.98	***dinJ***	1.39
icdC / iraM	0.71	*rpmG*	0.87	*lipA*	0.98	*yahA*	1.38
ydiY	0.71	*alaE*	0.87	*yciW*	0.97	*rpsLG-fusA-tufA*	1.38
tisB	0.71	*insEF-2*	0.87	*dps*	0.95	***dinD***	1.37

SI Table 3

MG1655, Type III experiment							
30 min		60 min		120 min		240 min	
Genes	**log$_2$ (Cy5/Cy3)**	Genes	**log$_2$ (Cy5/Cy3)**	Genes	**log$_2$ (Cy5/Cy3)**	Genes	**log$_2$ (Cy5/Cy3)**
yedEF	0.89	*hisDCBHAFI*	1.47	*araD / polB*	1.14	***recA***	1.5
yagH	0.73	*yqhD-dkgA*	0.99	***recA***	1.14	***recN***	1.35
trpD	0.68	*ackA-pta*	0.92	*yqhD-dkgA*	1.1	*frmAB*	1.3
nrdHI	0.6	*yedEF*	0.91	***recN***	1	*alaE*	1.06
yhiI	0.55	*frmAB*	0.73	***dinB***	0.85	*yqhD-dkgA*	1.03
frmA	0.51	*ndh*	0.72	***tisB***	0.82	*soxS-soxR*	0.98
ndh	0.5	*araB*	0.67	*yjeF*	0.82	*araD / polB*	0.97
		aceF	0.63	*mhpR / mhpA*	0.8	*mhpR / mhpA*	0.97
		kdpFABCDE	0.62	*frmAB*	0.72	***tisB***	0.96
		dadAX	0.61	*dadAX*	0.69	*yjhP*	0.84
		yagH	0.6	***dinG***	0.65	*sbmC*	0.77
		cusA	0.58	*mqo*	0.63	***dinD***	0.77
		araD / polB	0.54	*yfeO*	0.62	***umuCD***	0.72
		mqo	0.54	*lhgO*	0.62	***uvrB***	0.7
		yhiI	0.53	*soxS-soxR*	0.62	***yebG***	0.7
		recA	0.52	*yjhP*	0.62	*mocA*	0.7
				nemA	0.58	***uvrA***	0.69
				ruvA	0.57	*xisE*	0.67
				mtr	0.56	*ldhA*	0.67
				uvrA	0.54	*tdcR*	0.66
				uvrD	0.52	*yjeF*	0.65
						sulA	0.64
						sdaA	0.64
						ruvA	0.61
						evgA / emrK	0.61
						gntP	0.6
						cho	0.59
						fadD	0.58
						dinB	0.56
						grxA	0.56
						yghW	0.55
						dinG	0.54
						pspCDE	0.53
						dgkA	0.52
						lexA	0.52
						dadA	0.51
						nemA	0.51

SI Table 4: Sequences of primers used in this study

Creation and verification of *kefGB* null mutant:

Name of primer	Sequence (5' → 3)
kefGB-KO-F	CAGCATCCGCATATCCAGGCGGCGAAAATGCAGTTGCGCTCGCTGTGCCTGTTATTAAAGCTTATGCAGCGGAAAATGCAGC
kefGB-KO-R	CAATGGTAGCCCAAAACAGCGACTATACACAAAAACCATACCGGGAGGGGGTTCGAAACAGCTATGACCATGATTACG
kefGB KO check-F	GGCGTCGTTCTTGTTGC
kefGB KO check-R	AACAATCATAAGGTGCC
Apr Seq 1	CCTCGAAGATGGGCCACTT
Apr Seq 2	CTCGATCAGTCCAAGTGGC
Apr Seq 3	CGTTGATCTTCCTGCATCC
Apr Seq 4	GCATCGCATTCTTCGCATC

Verification of Keio collection mutants:

Name of primer	Sequence (5' → 3)
k1	CAGTCATAGCCGAATAGCCT
k2	TTGTCAAGACCGACCTGTCC
frmA-U	AAGGTCCTTACTCGAGTTCG
frmA-D	AGCATCTGCGACATGACTG
nemR-U	AAGAACTCTAATTGCTCCGC
nemR-D	CAATTTGCTCCGGACTATG
nemA-U	GAAGTGTGCGATCTGTCAG
nemA-D	GTAGGTCAGTTCAATCACCG
yqhD-U	AGCATTTCTCCAGCACTCTG
yqhD-D	TCCACAGCTTAGTGGTGATG

qPCR on cDNA to confirm expression levels:

Name of primer	Sequence (5' → 3)
frmA RT-F	AGAAGCAAACCATGAACACG
frmA RT-R	GGTTTGTCGTAGTCATTCGG
yqhD RT-F	AACCGGTTCAGAATCCAAC
yqhD RT-R	TTGGTAACATACTGTTCCACG
kdpA RT-F	GCTGTGAATACCGTTGAAGG

kdpA RT-R	CCATCACTTCACCAAAGGC
nemA RT-F	AACACGTACTTCTCTGCGC
nemA RT-R	GATCGGTACGATGGTTTGA
gloA RT-F	TACTCACTGGCGTTTGTTGG
gloA RT-R	CTTCACGGGTCACGTTACC
gloB RT-F	GGTGTATGGTCCACAAGAGAC
gloB RT-R	CCAATGTATCGTCAGGTAACG
kefG RT-F	GTGATTGTCTTTCAGCATCC
kefG RT-R	CATCGGGTAGCGATTCAG
kefF RT-F	GATCTGATCGTCTGGCAGC
kefF RT-R	CAGTTCAGCCCGCAGTAG
recA RT-F	GGTGTGATGTTCGGTAACC
recA RT-R	CGCCGTAGAAGTTGATACC
recN RT-F	CTACTGCAGGAAATGACCG
recN RT-R	AGCAATTGACCGCTGTTCG
uvrA RT-F	ATACCATTGAAGTGGTGGTTG
uvrA RT-R	CAGTTCACGCATACTGTAGC
lexA RT-F	GTCTGTTGCAGGAAGAGG
lexA RT-R	ACCGTTACGTACATCCTGAG
polA RT-F	CTGTGAACAACTGGAAGTGC
polA RT-R	CAAGGATGGTGACGTAGTTG
trkA RT-F	CAGCGTTAAACTCATCGAAC
trkA RT-R	GCTGGATCAATACCATCACC
topB RT-F	ACCGCATTAGTGGTCAACC
topB RT-R	AATAGCGACAATCAGAACGC
insF RT-F	GTTATTGGCTGGTCAATGTC
insF RT-R	CATTATCGTAGCAGCAACC

qPCR on cDNA analyze *recN* mRNA length:

<u>Primer pair 1</u>

recN 1	GGTCTTTGTCTCGGTGGT
recN 3	GTGCTGACCATGGATCTG

<u>Primer pair 2</u>

recN RT-F	CTACTGCAGGAAATGACCG (same as above)
recN RT-R	AGCAATTGACCGCTGTTCG (same as above)

<u>Primer pair 3</u>

recN 6	CACTACTGCGATCGTCTGG
recN 9	GCGTTGTTGGTGTAATGC

<u>Primer pair 4</u>

recN 10	CTGGCAATCCAGGTCATC
recN FL2	GCAGTTCTTTCGCATTCG

qPCR on IP-DNA:

recN

<u>Primer pair 1</u>

recN-RS-1	ACACATTAAGCACCAAGCTC
recN-RS-2	TTGCTGATGGTCAGTTGTG

<u>Primer pair 2</u>

recN-RS-5	TACCATCCAGATTGCTGAAG
recN-RS-6	TGGTAATACTGTGGCAATGC

recA

<u>Primer pair 1</u>

recA-RS-1	CCTTGTGGCAACAATTTCTAC
recA-RS-2	GTTTGTTTTCGTCGATAGCC

<u>Primer pair 2</u>

recA-RS-5	TATCAACTTCTACGGCGAAC
recA-RS-6	TTCTTCTCGATCTCTTTCGC

frmA

Primer pair 1

frmA-RS-1	GACGACACTATTGAACTGGTTC
frmA-RS-2	GCAACGTCAATTTCAACG

Primer pair 2

frmA-RS-3	CGACCATACCTTTGAATGC
frmA-RS-4	GTGACCAACTGGAATGGAC
